# Multi-omics analysis identifies osteosarcoma subtypes with distinct prognosis indicating stratified treatment

**DOI:** 10.1038/s41467-022-34689-5

**Published:** 2022-11-23

**Authors:** Yafei Jiang, Jinzeng Wang, Mengxiong Sun, Dongqing Zuo, Hongsheng Wang, Jiakang Shen, Wenyan Jiang, Haoran Mu, Xiaojun Ma, Fei Yin, Jun Lin, Chongren Wang, Shuting Yu, Lu Jiang, Gang Lv, Feng Liu, Linghang Xue, Kai Tian, Gangyang Wang, Zifei Zhou, Yu Lv, Zhuoying Wang, Tao Zhang, Jing Xu, Liu Yang, Kewen Zhao, Wei Sun, Yujie Tang, Zhengdong Cai, Shengyue Wang, Yingqi Hua

**Affiliations:** 1 Department of Orthopedic Oncology, Shanghai Bone Tumor Institute, Shanghai General Hospital, Shanghai Jiao Tong University School of Medicine, Shanghai, 200080 PR China; 2 National Research Center for Translational Medicine (Shanghai), State Key Laboratory of Medical Genomics, Shanghai Institute of Hematology, Ruijin Hospital, Shanghai Jiao Tong University School of Medicine, Shanghai, 200025 PR China; 3 Key Laboratory of Cell Differentiation and Apoptosis of National Ministry of Education, Department of Pathophysiology, Shanghai Jiao Tong University School of Medicine, 200001 Shanghai, PR China; 4 Department of Pathology, Shanghai General Hospital, Shanghai Jiao Tong University School of Medicine, Shanghai, 200080 PR China

**Keywords:** Cancer genomics, Sarcoma, Tumour biomarkers

## Abstract

Osteosarcoma (OS) is a primary malignant bone tumor that most commonly affects children, adolescents, and young adults. Here, we comprehensively analyze genomic, epigenomic and transcriptomic data from 121 OS patients. Somatic mutations are diverse within the cohort, and only TP53 is significantly mutated. Through unsupervised integrative clustering of the multi-omics data, we classify OS into four subtypes with distinct molecular features and clinical prognosis: (1) Immune activated (S-IA), (2) Immune suppressed (S-IS), (3) Homologous recombination deficiency dominant (S-HRD), and (4) *MYC* driven (S-MD). *MYC* amplification with HR proficiency tumors is identified with a high oxidative phosphorylation signature resulting in resistance to neoadjuvant chemotherapy. Potential therapeutic targets are identified for each subtype, including platinum-based chemotherapy, immune checkpoint inhibitors, anti-*VEGFR*, anti-*MYC* and PARPi-based synthetic lethal strategies. Our comprehensive integrated characterization provides a valuable resource that deepens our understanding of the disease, and may guide future clinical strategies for the precision treatment of OS.

## Abstract (web summary)

Osteosarcoma survival rates have been largely unchanged for several decades, and treatment response is variable. Here, the authors analyzed genomic, transcriptomic and epigenomic data from 121 osteosarcoma patients and identify subtypes related to treatment outcome.

## Introduction

Cancers of bone and joints rank as the third leading cause of cancer death among children, adolescents and young adults^1^. As the most frequent bone neoplasia, osteosarcoma (OS) accounts for approximately 35% of primary malignant bone tumors^2^. Neoadjuvant chemotherapy and advances in surgical techniques have led to a dramatic improvement in overall survival rates to nearly 70%^3^. However, further progress in 5-year survival has been appreciably unchanged over the past four decades and therapeutic approaches are urgently needed^4^.

Previous pioneering studies over the past decade have expanded our understanding of the molecular basis of OS. As previously reported, the most frequently mutated genes in OS were *TP53* and *RB1*^5–9^. Multiple regions of somatic copy number alterations have been uncovered, including amplification of *MYC*, *CCNE*, and *AKT*, as well as deletion of *TP53*, *RB1* and *PTEN*^10–12^. However, those studies mainly focused on just single or two omics techniques, providing limited knowledge of the integrated biological underpinnings of this disease. Comprehensively integrated diverse omics data in conjunction with clinical information are therefore urgently needed.

Here, we present a large cohort of 121 OS patients from Shanghai General Hospital (SGH-OS cohort), using hybrid-capture DNA sequencing, array-based DNA copy number analysis, DNA methylation profiling and mRNA sequencing. Through integrating multi-omics data, we obtained a comprehensive genomic, epigenomic and transcriptomic landscape of OS and uncovered four distinct molecular subtypes with varied oncogenic factors. We explored the diversity of clinical outcomes and the targeted intervention of each subtype. Our study provides insight into the biology of this disease, and potential avenues for future precision medicine approaches within OS.

## Results

### Clinical and molecular features of the SGH-OS cohort

The SGH-OS cohort contains the molecular and clinical data of total 121 primary OS patients with mostly Enneking IIB and III stage (Supplementary Fig. 1A). All samples were re-reviewed and confirmed by two pathological experts individually based on the World Health Organization (WHO) classification system (2020) (Supplementary Fig. 1B). Specifically, 102 patients with surgically resected tumor species and matched white blood cells were profiled by whole-exome sequencing (WES). The transcriptomes of 96 patients were characterized by RNA sequencing (RNA-seq). DNA methylation and copy number profiles were generated from 114 patients using an Illumina Infinium EPIC BeadChip array (850 K). In addition, copy number alterations in 50 patients were further validated by the Affymetrix OncoScan microarray. The initial diagnosis age was between 6 to 67 years old, and 63 patients (52.1%) were pediatric or adolescent (under 18 years old, Supplementary Fig. 1C). Of those, 58 (47.9%) were female and 63 (52.1%) were male. The median follow-up was 34.9 months and the overall survival rate was explored (Supplementary Fig. 1D). Among all 121 participants, 61 patients (50.4%) had distant metastatic events, 35 patients (28.9%) suffered local recurrence, and 43 patients (35.5%) had died by the last follow-up. The influence of age, sex, tumor position, Enneking stage and pathological type on clinical prognosis was analyzed (Supplementary Fig. 1E–I). Other basic clinical information of the SGH-OS cohort is available in Supplementary Data 1.

### Mutational landscape of OS patients

A total of 107 tumors and 105 matched white blood cell samples from 102 OS patients were subjected to WES. Overall, we defined a diverse range of cancer genes mutated in OS. The tumor versus white blood cell comparison identified 6,381 mutated genes (Supplementary Fig. 2A–D, Supplementary Data 2). The median number of non-silent variants per sample was 33 (Supplementary Fig. 2E), which was relatively lower than that in pan-cancer published in TCGA (Supplementary Fig. 2F). The median tumor mutational burden (TMB) of SGH-OS was 1.4 per megabase (Mb), and there were no significant differences in overall TMB between adolescent, young adult and elderly OS (Supplementary Fig. 2G).

Among cancer-related signaling pathways with potential therapeutic targets, the *RAS, NOTCH, WNT, Hippo, PI3K*, and *MYC* pathways were predominantly affected (Supplementary Fig. 3A). In addition, the SGH-OS cohort showed relatively lower frequencies of mutations in *TP53, RB1, ATRX, MDM2* and *CCNE1* than those previously reported^13^, while the prevalence rates of *CHD3, GNAS, CIC* and *H3F3A* mutations were slightly higher in our cohort (Supplementary Fig. 3B). In particular, *TP53* (12 tumors, 11.2%) was identified as a significantly mutated gene (SMG) determined by MutSigCV algorithms (*q* value < 0.01), which was consistent with previous studies (Supplementary Fig. 3C, Supplementary Data 3).

**Fig. 1:**
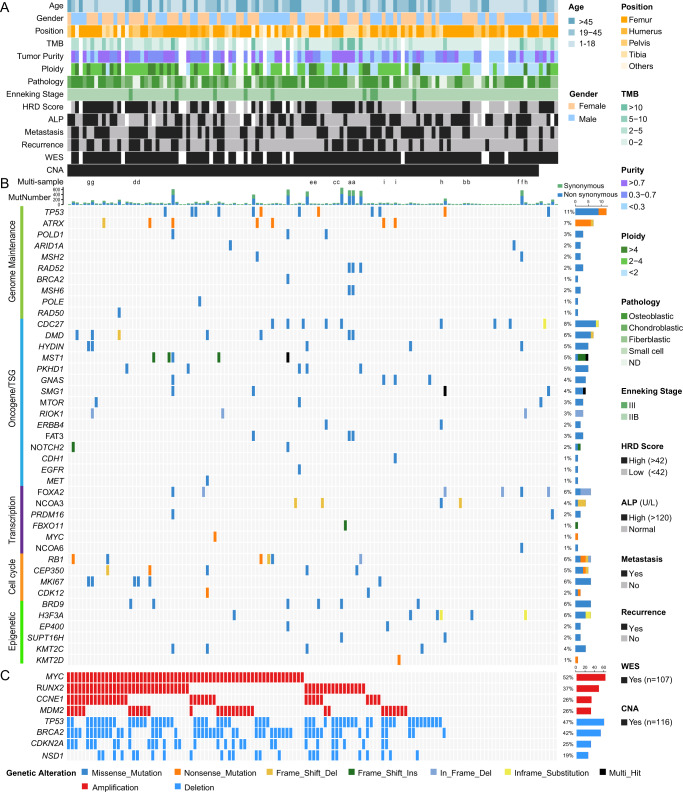
Genomic landscape of OS. Genetic profile of SGH cohort patients. Each column corresponds to one sample (128 samples are displayed with 9 replicates). **A** Clinical information of age, sex, position, metastasis, recurrence, pathological classification, ALP (high: >120 U/ml) and clinical Enneking stages. Genetic information included TMB, HRD score (high: >42), ploidy and tumor purity. **B** Somatic mutated genes that were associated with genome maintenance, oncogene/tumor suppressor gene (Oncogene/TSG), cell cycle, epigenetic and transcriptional regulation; **C** Cancer-related genes located in significant CNA peaks identified by GISTIC 2.0 with *q* value <0.25. The number and percentage of mutations and CNAs for each of the genes are shown on the right.

The clinical features and mutation profile of genes related to genome maintenance, oncogene/tumor suppressor gene (Oncogene/TSG), cell cycle, epigenetic and transcriptional regulation are shown in Fig. 1A, B and Supplementary Data 2. We compared the mutated genes (>3 cases) in the SGH-OS cohort with 3 curated cancer driver gene datasets^14–16^. This yielded 22 somatic mutated genes (Supplementary Fig. 3D, Supplementary Data 4), which may be potential oncogenic drivers in OS. In addition, we discovered several other mutations of *KMT2B* (2.8%) and *RARA* (1.9%) in Chinese OS patients. We further analyzed the germline mutations of the SGH-OS cohort (Supplementary Data 5). There were no significant differences in overall germline mutation load between adolescent, young adult and elderly OS (Supplementary Fig. 2H). Functional enrichment analysis revealed that germline mutated genes were primarily enriched in metabolism-related KEGG pathways (Supplementary Fig. 2I).

To further address the potential driving mutational processes of OS, we applied non-negative matrix factorization (NMF)^17^ and identified four predominant signatures similar to COSMIC^18^ 1, 3, 4 and 15 signatures (Supplementary Fig. 3E). Among those, defects in DNA double-strand break (DNA-DSB) repair by homologous recombination (HR) not only enable cancer cells to accumulate genomic alterations that contribute to their aggressive phenotype, but may also serve as a vulnerable drug target, offering the possibility of personalized therapies^19–21^.

### CNA analysis reveals clinical implications for OS patients

**Fig. 2:**
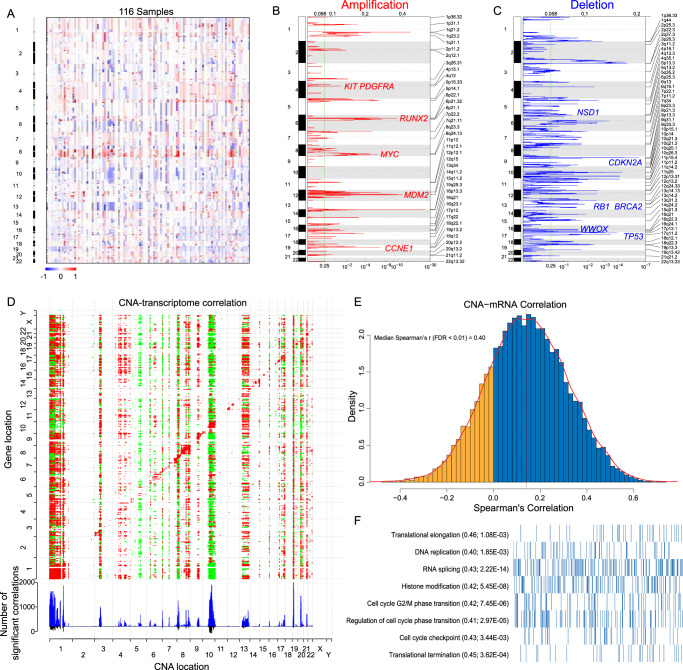
Copy number alteration profiles of OS. **A** Heatmap of the CNAs of 116 OS tumor samples: red and blue represent copy number gain and loss, respectively. The x axis indicates the 116 tumor samples. **B** Genome-wide recurring focal amplifications with GISTIC 2.0 FDR q values on the bottom. Peaks were annotated with candidate driver oncogenes in red. **C** Genome-wide recurrent focal deletions with GISTIC 2.0 FDR q values on the bottom. Candidate driver tumor suppressors within deletion peaks are labeled in blue. **D** Correlations of CNAs to mRNA expression with *cis* and *trans* effects. Significant positive (red) and negative (green) correlations (see “Methods”, FDR < 0.01, Spearman’s correlation) between CNAs and mRNA are indicated in the upper panel. The X-axis and Y-axis are ordered by chromosomal location. The blue bars in the bottom panel represent the number of specific significant correlations, while the black bars indicate the number of common significant correlations. **E** Distribution of Spearman’s correlation between CNAs and mRNA. CNAs and mRNAs were positively correlated for most (78.9%) CNA-mRNA pairs. The median Spearman’s coefficient of significant correlations (FDR < 0.01) was 0.40. **F** Significantly enriched functions of genes with significant correlations between CNAs and mRNA. The median correlation coefficient is shown in parentheses, followed by the FDR adjusted *P* value. Genes in each item (bars on the x-axis) were sorted by correlation coefficients from low to high, with blue and yellow indicating positive and negative correlations, respectively.

A total of 116 OS samples from 114 patients were employed for somatic copy number alteration (SCNA) analysis based on the Illumina Infinium EPIC BeadChip (850 K) array (Fig. 2A). GISTIC 2.0 analysis of recurrent CNAs revealed 39 significantly amplified and 53 deleted regions (q value <0.25). The frequent copy number gains with potential biological implications were in chromosomes 1q, 4q, 6p, 8q, 12q, 14q, and 19q, and the losses were in chromosomes 5p, 9p, 13q, and 17p. Recurring amplifications included *MYC* (8q24.13, 52%), *RUNX2* (6p21.1, 37%), *PDGFRA* (4q12, 35%), *CCNE1* (19q12, 26%) and *MDM2* (12q15, 26%) (Fig. 2B, Supplementary Data 6). Recurrent deletions included the key tumor suppressors *TP53* (17p13.1, 47%), *RB1* (13q14.13, 42%), *CDKN2A*/*CDKN2B* (9p21.3, 25%), and *NSD1* (5q35.2, 19%) (Fig. 2C, Supplementary Data 6). In addition, CNAs in 50 patients were validated using a high-resolution OncoScan array with the same DNA specimen in parallel to the EPIC 850 K array and similar patterns of SCNAs were displayed using these two different approaches (Supplementary Fig. 4A).

To determine the consequences of CNAs on mRNA abundance, we explored the cis and trans effects between CNAs and mRNA abundance (Fig. 2D). A total of 17,242 CNA-mRNA pairs were analyzed with 2,522 in cis displaying significant correlation (FDR < 0.01, median Spearman’s *r* = 0.40) (Fig. 2E, Supplementary Data 7), Interestingly, there was no broad cis-regulatory effects of CNA on mRNAs, functional enrichment analysis revealed that these genes were mainly enriched in cell cycle regulation, DNA replication, translation regulation and histone modification (Fig. 2F), suggesting that those biological processes may largely be regulated by aberrant CNAs. We also noted the most prominent CNAs with trans effects on chromosomes 1q, 2p, 8q, 11p, 19q and 21p. Among them, 8q, containing the *MYC* oncogene, was the predominantly amplified region, which indicated that *MYC* amplification had both cis- and trans-regulatory effects on its targets during the carcinogenesis of OS.

Deletion of the cell cycle regulator *CDKN2A*/*CDKN2B* was associated with poor clinical prognosis in patients in our cohort (Supplementary Fig. 4B). As decreased *CDKN2A*/*CDKN2B* levels further result in elevated *CDK4*/*cyclin D* activity, these patients may benefit from CDK4 inhibitors^22^. Another significant deletion peak at 5q35.2 containing *NSD1* merits further exploration. As an H3K36 di-methyltransferase, *NSD1*, which was lost in 22 out of 116 cases, is implicated as a tumor suppressor gene mutated frequently in a variety of carcinomas, and associated with global DNA hypomethylation^23–26^. We detected the expression level of NSD1 in an individual tissue microarray containing 49 OS cases and NSD1 deletion patients in the SGH-OS cohort by immunohistochemistry. The results confirmed that the expression level of NSD1 in OS was positively correlated with H3K36me2 modification. In addition, the expression levels of NSD1 and H3K36me2 signals were undetectable in NSD1 deletion patients (Supplementary Fig. 4C). Therefore, we reasoned that the epigenetic changes induced by *NSD1* deletion might be involved in OS oncogenesis.

### Patient subtyping by different molecular dimensions

**Fig. 3:**
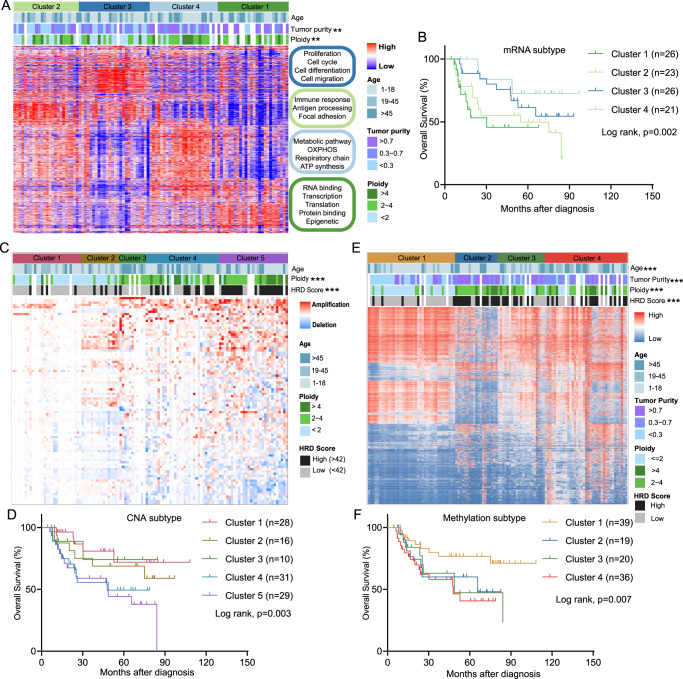
Single platform features and the corresponding clinical prognosis. **A** Transcriptional clustering based on the top 10% most variable genes (1820) across 101 samples. Each column represents one sample and rows indicate genes. **B** Kaplan–Meier curves for overall survival based on transcriptional clusters (log-rank test). **C** Copy number clustering based on SCNAs identified by GISTIC 2.0 in 116 samples. Each column represents one sample, and rows indicate CNA peaks. **D** Kaplan–Meier curves for overall survival based on CNA clusters (log-rank test). **E** DNA methylation clustering based on the top 8000 variably methylated CpG sites in 116 samples. Each column represents one sample and rows indicate CpG sites. **F** Kaplan–Meier curves for overall survival based on DNA methylation clusters (log-rank test). Asterisks define significance levels (*p < 0.05; **p < 0.01; ***p < 0.001; ****p < 0.0001).

We explored the molecular subtyping using each individual omics dataset. Unsupervised consensus clustering based on the top 10% most variably expressed coding genes was applied to identify transcriptomic subtypes of OS. Based on the “elbow” point in the relative change in area under the consensus distribution function (CDF) curve, we classified 101 tumors into 4 distinct clusters (Supplementary Fig. 5A–F). These four clusters consisted of the following: (1) mRNA Cluster 1 was characterized by overexpression of transcription- and translation-related genes; (2) mRNA Cluster 2 was dominated by the highest level of adaptive immune response and immune signaling pathway related gene expression; (3) mRNA Cluster 3 featured activation of the transforming growth factor-β (*TGF-β*) signaling pathway and the downstream *PI3K/AKT* and *MAPK* pathways; and (4) mRNA Cluster 4 harbored the signature of genes involved in mitochondrial energy transduction and ATP synthase activity (Fig. 3A). Candidate targets identified within each cluster indicated the potential applications of biomarker-driven precision therapies. Moreover, the transcriptomic clusters significantly differed in clinical prognosis (log rank, *p* = 0.002, Fig. 3B).

CNAs not only serve as “drivers” for boosting tumor cell growth but also reflect the innate biological characteristics of tumors^27^. Unsupervised hierarchical clustering analysis of CNAs yielded five clusters with varied ploidy and HRD scores (Fig. 3C), and the potential prognostic value of CNA subtype strategies was investigated (Fig. 3D). CNA Cluster 1 was nearly devoid of significant CNAs, featuring lower tumor purity and aneuploidy, corresponding to immune activation and favorable clinical prognosis (Supplementary Fig. 5G). Similar to CNA Cluster 1, CNA Cluster 2 consisted mainly of low purity tumors, distinguished by more frequent 1q31.1(*NR5A2*, *PTGS2, RGS1*), 3q36.1 (*BCHE*), 5p14.1 (*PRDM9*), 11p12 (*API5*), and 21q11.2 (*RBM11, LIPI*) amplification and relatively worse clinical prognosis (Supplementary Fig. 5H). CNA Cluster 3 exhibited chromosome 12 amplification (including the *MDM2* and *CDK4* loci) in a manner largely cooccurring with *CDKN2A* deletion (Supplementary Fig. 5I). As *CDKN2A* deletion activates *CDK4/CDK6*, leading to higher RB phosphorylation and driving cell cycle progression^28^, CDK4/6 inhibitors may be a therapeutic option for CNA Cluster 3 patients. CNA Cluster 4 and Cluster 5 exhibited the highest burden of CNAs and the worst clinical prognosis, featuring deletion of *TP53* and *BRCA2*. This suggested DNA maintenance defects in those patients who may respond to DNA-damaging agents.

Unsupervised clustering of OS using the top 8000 most variable CpG loci that were hypermethylated in at least 5% of tumors identified 4 clusters. Tumor purity, ploidy and HRD score differed significantly across different DNA methylation subgroups (Kruskal–Wallis test, *p* < 0.001) (Fig. 3E). The overall survival between each cluster was analyzed (Fig. 3F). Of particular interest was methylation Cluster 1 patients, with more young patients having the best clinical prognosis, while elderly patients with OS were more enriched in methylation Cluster 4, with poor survival. Correlations between DNA methylation and gene expression were explored (Supplementary Data 8). Eight representative frequently hypermethylated genes are shown in Supplementary Fig. 5K. These genes displayed reduced mRNA expression, which was negatively correlated with promoter hypermethylation.

### Integrative multi-omics analysis stratifies clinically relevant OS subtypes

**Fig. 4:**
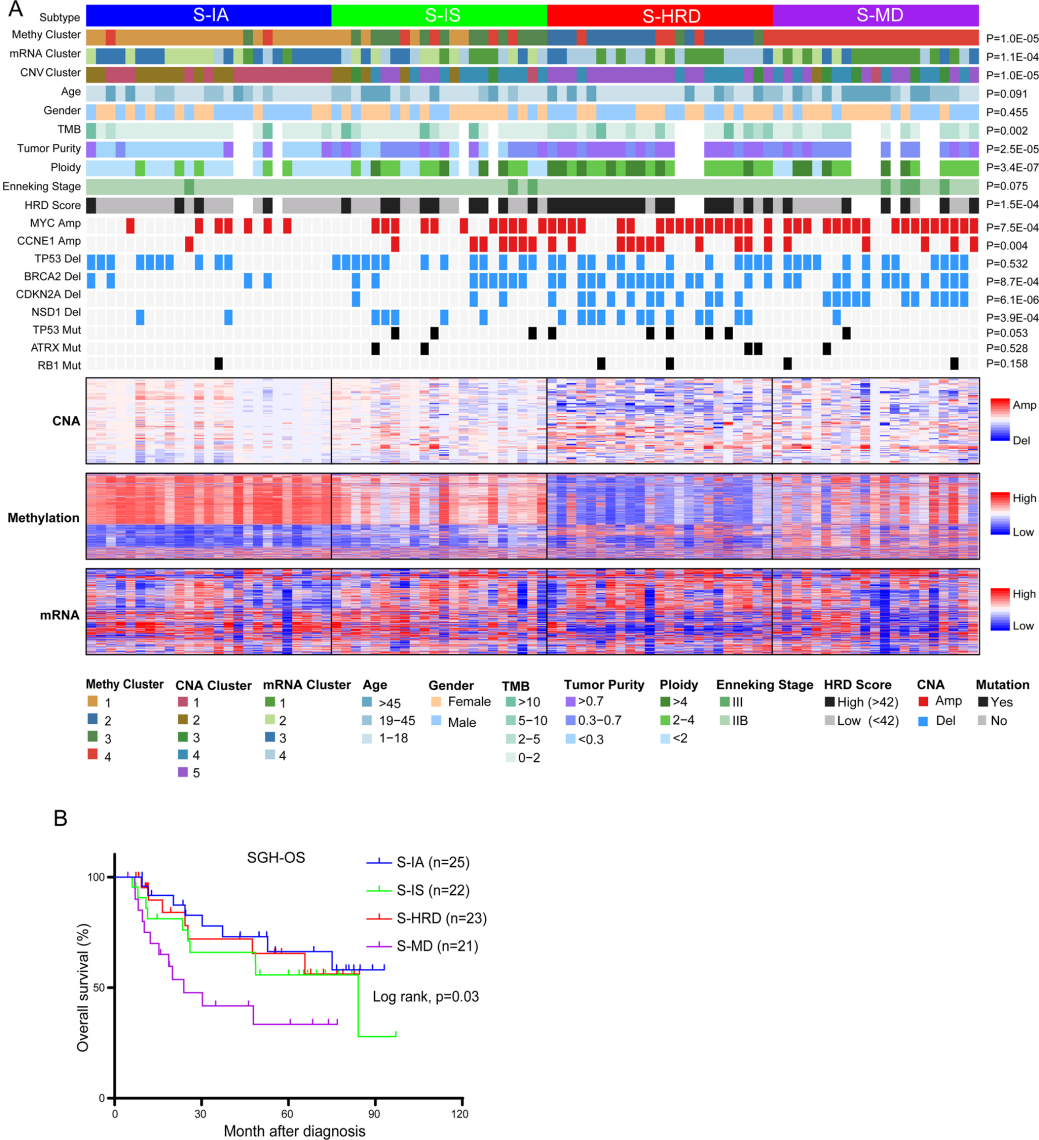
Integrative subtypes with distinct molecular features and varied clinical prognosis. **A** Integrative clustering of 91 patients. Top, left to right: immune-activated subtype (S-IA, iCluster1), immune-suppressed subtype (S-IS, iCluster2), HRD dominant subtype (S-HRD, iCluster3) and MYC driven subtype (S-MD, iCluster4). Single platform clustering results: DNA methylation cluster, mRNA cluster and CNA cluster. Basic clinical features: age, gender and Enneking stage. Genetic changes: tumor purity, ploidy, HRD score, somatic mutations and CNAs. Bottom, Heatmaps organized by integrative clustering for copy number, DNA methylation, and mRNA expression. **B** Kaplan–Meier curves for overall survival based on integrative subtypes (log-rank test).

The acquisition of cancer hallmarks requires molecular alterations not only at the transcriptome level but also at multiple other levels, including the genome and epigenome^29,30^. To determine multi-omics data in OS subtyping, iCluster, a joint multivariate regression algorithm^31,32^, was applied to reconcile and cluster these disparate data from multiple platforms simultaneously. This unsupervised integrative clustering ultimately defined four distinct subtypes within 91 patients (Fig. 4A and Supplementary Fig. 6A).

The majority of the single platform clusters concentrated preferentially in one of the four integrated iClusters with *p* < 0.01, lending confidence that the integrative subtyping strategies captured the main features of each platform (Fig. 4A). Consistent with Fig. 4A, single sample GSEA further demonstrated distinct molecular features among the four subtypes (Supplementary Fig. 6B). Subtype specific somatic mutations and copy number changes were also explored (Supplementary Fig. 6C, D, Supplementary Data 9). Based on the molecular characteristics described below, we designated these four clusters as immune-activated subtype (S-IA, iCluster1), immune-suppressed subtype (S-IS, iCluster2), HRD dominant subtype (S-HRD, iCluster3) and MYC driven subtype (S-MD, iCluster4).

iCluster1 (*n* = 25) was characterized by the lowest tumor purity, proliferation activities and high immune responses (Fig. 4A, Supplementary Fig. 6B). DNA methylation Cluster 1 and CNA Cluster 1–2 with favorable prognosis were mainly enriched in this subtype. The iCluster1 subtype exhibited low frequencies of *MYC*, *CCNE1* amplification and *CDKN2A* deletion. iCluster1 tumors also had specific changes in mRNA expression, including overexpression of *BANK1* (Supplementary Fig. 7A), a crucial regulator acting as a tumor suppressor involved in both B-cell mediated humoral immunity and cellular immunity^33,34^. In addition, the expression levels of the core osteogenic transcription factor *RUNX2* were significantly different among different subtypes, with the lowest expression in S-IA, suggesting that immune-activated OS has relatively lower osteogenic activity. (Supplementary Fig. 7B).

iCluster2 (*n* = 22) had relatively higher tumor purity and aneuploidy than iCluster1. GSEA revealed the activation of adipogenesis- and fatty acid metabolism-related pathways in iCluster1 compared to iCluster2 (Supplementary Data 10 and Supplementary Fig. 7C). This corresponded to the focal amplification of the 7q21.12 locus in iCluster1, which encodes the fatty acid scavenger receptor *CD36* (Supplementary Fig. 6D). In addition, cell cycle-related pathways, including E2F targets and the G2/M checkpoint, were downregulated in iCluster1 (Supplementary Fig. 7D).

iCluster 3–4 was more likely driven by proliferative signaling including the cell cycle, *MYC*, mTOR and Hedgehog pathways, indicating the high proliferative potential of these two subtypes (Supplementary Fig. 6B). iCluster3 (*n* = 23) was characterized by the highest tumor purity and genomic instability. Tumors in this subtype contained most of those in DNA methylation Cluster 2 and CNA Cluster 5 with the lowest immune responses. This subtype was also associated with *NSD1* deletion and overexpression of proliferative genes, such as *CCL28, HUNK, ZFHX4, GRHL3* and *CHAF1B* (Supplementary Fig. 7A).

iCluster 4 (*n* = 21) was identified as the most malignant subtype, with a 5-year survival rate lower than 40%. Methylation Cluster 4 was heavily enriched in this subtype. The prominent features of this subtype were *MYC* amplification, *mTOR* signaling pathway activation, and low immune responses. GSEA demonstrated the activation of *MYC* targets in iCluster4 compared with others (Supplementary Fig. 7E).

We further tested the clinical relevance of the integrative subtyping, and the results indicated that the four subtypes significantly differed in clinical prognosis (log rank, *p* = 0.03, Fig. 4B). After stratifying patients according to Enneking stage, integrative subtypes were still strongly correlated with patient prognosis in Enneking stage IIB patients (Supplementary Fig. 7F), supporting the superior prognostic power of molecular features within our integrated molecular subtypes.

### *MYC* amplification with HR proficiency was associated with OXPHOS activation and poor prognosis

**Fig. 5:**
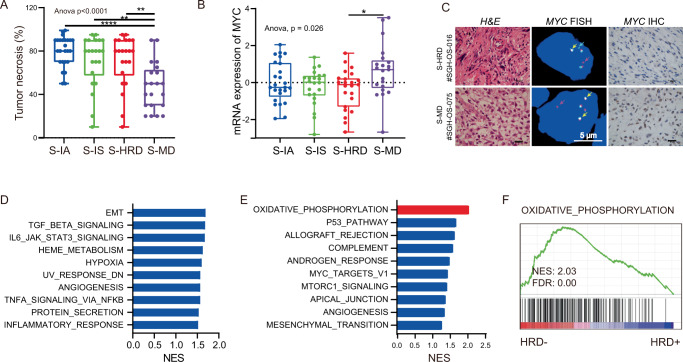
*MYC* amplification with HR proficiency was associated with OXPHOS activation and poor prognosis. **A** Comparison of the tumor necrosis rate among integrative subtypes. The statistical analysis was made by ANOVA with Tukey’s multiple comparisons test. (*P* < 0.0001). **B** Gene expression level of *MYC* in each subtype. The Student’s *t* test was adopted in significant difference analysis between S-HRD and S-MD. **C** Rearrangements of *MYC* tested by FISH and expression level of *MYC* protein measured with IHC. 3 times repeated independently. **D** GSEA between *MYC*-amplified and *MYC* unamplified OS. **E**, **F** GSEA between *MYC*-amplified HR proficiency and *MYC*-amplified HR deficiency OS.

We identified that patients in S-MD subtype displayed the lowest tumor necrosis rate corresponding to the worst clinical prognosis (Fig. 5A). More importantly, the expression level of *MYC* in S-MD was significantly higher than that in S-HRD (Student’s *t* test, *p* < 0.05, Fig. 5B). These findings were further validated, indicating that *MYC* was mainly expressed in the nucleus of tumor cells compared with stromal cells (Fig. 5C). The difference between *MYC* expression could partially be attributed to *Hunk* overexpression in S-HRD, which is inhibitory to *MYC* expression (Supplementary Fig. 7A)^35^. We speculated that the malignant biological behavior caused by *MYC* overexpression was the leading cause for the poor prognosis of S-MD.

Considering that *MYC* amplification is one of the most important genetic features in OS leading to a relatively poor prognosis (Supplementary Fig. 7G), we compared the transcriptomic profiles between tumors with or without *MYC* amplification. This revealed that epithelial-mesenchymal transition, *JAK/STAT3* and *TGF-β* signaling pathways were upregulated in *MYC*-amplified OS (Fig. 5D). These findings further supported the crucial roles of *MYC* amplification in the malignant phenotype of OS.

By dividing *MYC* amplification OS patients into two groups according to the HRD score, we observed that the clinical prognosis of *MYC* amplification with HR proficiency was significantly worse than that with HR deficiency (Supplementary Fig. 7H). GSEA indicated that *MYC*-amplified OS with HR proficiency exhibited significant OXPHOS activation compared to that with HR deficiency (Fig. 5E, F). These findings revealed that mitochondria‐related OXPHOS was positively associated with unfavorable prognosis, which may be associated with CDDP chemoresistance.

### Immune landscape of OS

With the rapid advancement in genomics, the treatment of OS, particularly for relapsed and metastatic patients, has evolved away from conventional chemotherapy toward immune-based strategies^36–41^. Accordingly, we characterized the immune microenvironment in OS.

**Fig. 6:**
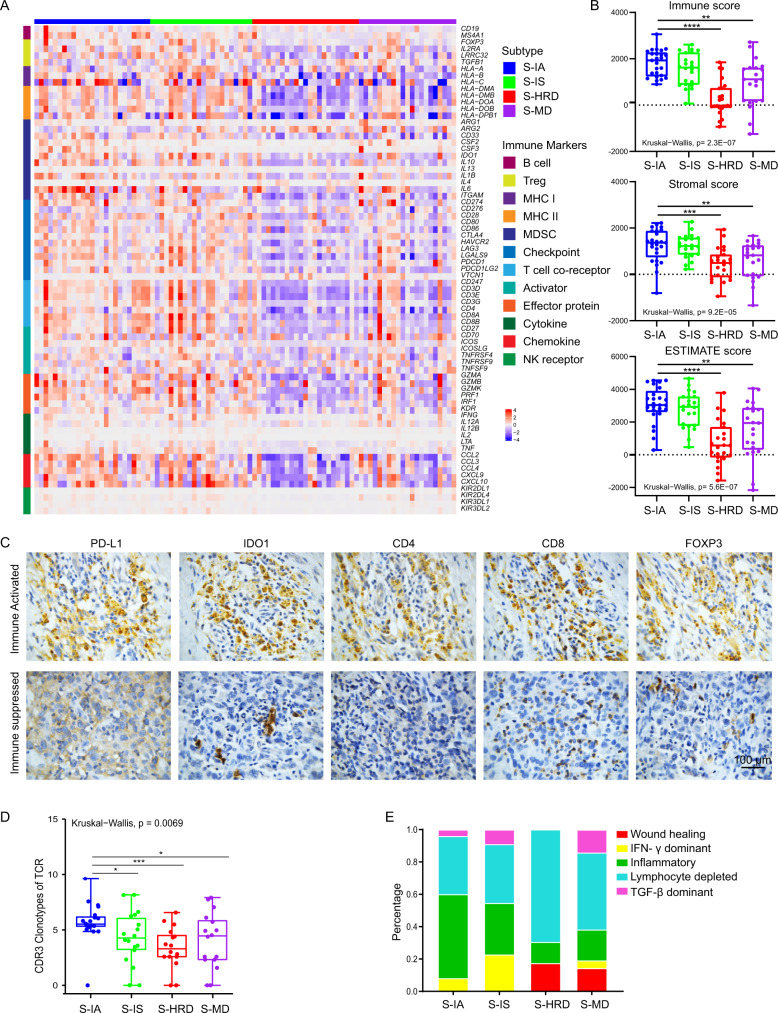
Immune landscape of OS. **A** Heatmap of 72 curated immune markers within OS patients. The integrative subtype information of each patient is annotated at the top. **B** The immune score, stromal infiltration score and ESTIMATE score among each integrative subtype. The statistical analysis was made by Kruskal–Wallis test. **C** Immunohistochemistry of *CD4*, *CD8*, IDO1, FOXP3 and PD-L1 between iCluser 1–2 and iCluster 3–4. **D** CDR3 clonotypes of TCRs in each subtype. *P* = 0.0069, the statistical analysis was made by Kruskal–Wallis test. **E** Distribution of immune subtypes within integrative subtypes. Asterisks define significance levels (*p < 0.05; **p < 0.01; ***p < 0.001; ****p < 0.0001).

As shown in Supplementary Fig. 6B, the immune responses were higher in iCluster 1–2 than in iCluster 3–4. iCluster 1–2 were high in the interferon alpha, interferon gamma, inflammation, *CTLA-4, IL-17* signaling pathways and others related to the immune response. To further investigate the immune microenvironment of OS, we explored the gene expression levels of 72 curated immune surface markers that encompass different immune cell populations in each subtype^42–44^. In line with Supplementary Fig. 6B, iCluster 1-2 exhibited higher expression of the immune markers corresponding to immune reactions than iCluster 3–4 (Fig. 6A). Consistent with these finding, the immune and stromal scores were relatively higher in iCluster 1–2 (Fig. 6B).

We next estimated the relative percentage of 22 types of tumor‐infiltrating immune cells (TIICs) using the Cell-type Identification by Estimating Relative Subsets of RNA Transcripts (CIBERSORT) algorithm as previously reported^45^ (Supplementary Fig. 8A, Supplementary Data 11). Significant differences in *CD8*+ T cells, type 2 macrophages, naive *CD4* T cells and resting memory *CD4* T cells between iCluster 1-2 and iCluster 3–4 were observed (Kruskal–Wallis test, *p* < 0.05, Supplementary Fig. 8B). Immunohistochemistry further confirmed that immune-related markers, including CD4, CD8, IDO1, FOXP3 and PD-L1, were expressed at relatively higher levels in iCluster 1-2 (Fig. 6C). Moreover, we noted significant depletion of gamma delta T cells and augmentation of monocytes in iCluster1 compared with iCluster2 (Wilcoxon rank sum test, *p* < 0.05, Supplementary Fig. 8B), indicating a transformation of the immune state from activation to suppression.

The T-cell receptor (TCR), reflected by the most variable complementarity-determining region 3 (*CDR3*) region, has a critical role in antigen recognition^46,47^. To investigate tumor-reactive T-cell clones, we characterized the repertoire of tumor-infiltrating T cells inferred by TRUST^47^. Strikingly, although iCluster 1–2 both displayed stronger immune responses, we unexpectedly found that the number of CDR3 calls in iCluster1 was significantly higher than that in iCluster2 (Wilcoxon rank sum test, *p* < 0.05, Fig. 6D). Collectively, these results indicated that tumors in iCluster1 were immune-activated (S-IA), while those in iCluster2 were immune suppressive or exhausted (S-IS).

To further characterize the intratumoral immune states, we inferred the immune subtypes defined previously for each OS patient^48^. The immune categories varied largely across different iClusters (Fig. 6E). iCluster1 was rich in immune subtype C3 (Inflammatory), which was defined by a low level of cell proliferation and the best prognosis. C2 (*IFN-γ* dominant), with a high cell proliferation rate and the highest M1, was enriched more in iCluster2. In addition, iCluster2 had less C3 (inflammatory) and more C6 (*TGF-β* dominant), together resulting in worse outcomes than iCluster1. iCluster3 and iCluster4 were primarily dominated by C4 (lymphocyte depleted), further indicating that these two subtypes belong to cold tumors, and these findings were concordant with the clinical prognosis of each subtype (Figs. 4B and  6E).

### Subtype specific targeted therapy strategies

**Fig. 7:**
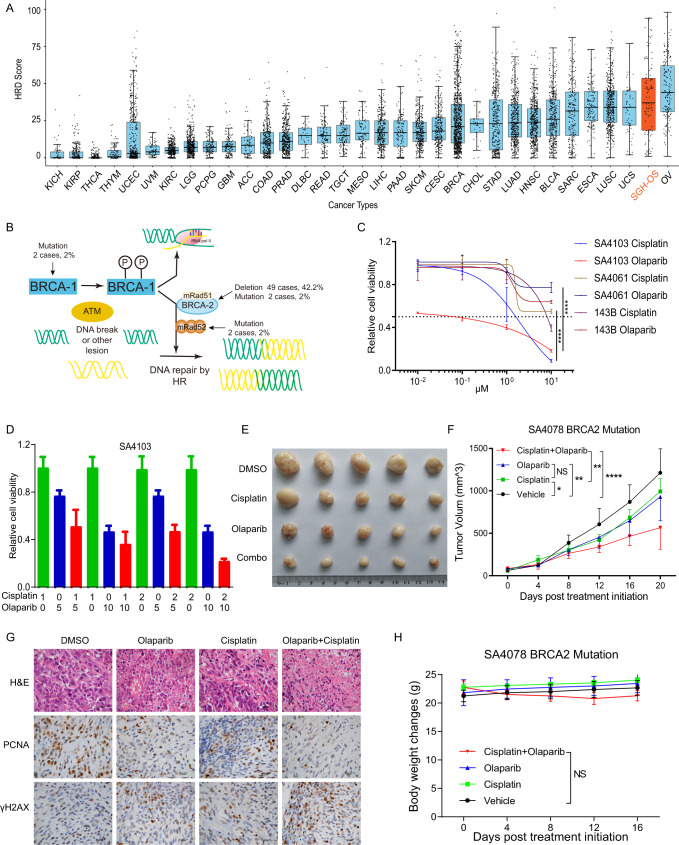
Synthetic lethality of olaparib plus cisplatin in HRD-positive OS in vitro and in vivo. **A** Distribution of HRD scores within and across 34 cancer types. The SGH-OS cohort is labeled (orange, *n* = 107). **B** Schematic model of the HR and the genomic alterations in the key elements. **C** Dose response curves and IC50 values of HRD-positive (SA4103), HRD-negative (SA4061) and conventional OS cell lines after 48 h of exposure to the cisplatin and PARP inhibitor olaparib. ****p < 0.0001. Data are shown as mean ± SD. *n* = 3 independent experiments. *P* values are derived from two-sided *t* test. **D** Synthetic lethality of olaparib plus cisplatin in the SA4103 cell line. Data are shown as mean ± SD. *n* = 3 independent experiments. The statistical analysis was made by ANOVA with Tukey’s multiple comparisons test. Tumor gross specimen (**E**) and tumor growth curves (**F**) in a patient-graft xenograft model treated with olaparib and cisplatin individually or in combination. (*n* = 5 mice per group). Data are represented as the mean ± SD, *P* values are derived from two-way ANOVA. *p < 0.05; **p < 0.01; ***p < 0.001. **G** H&E and immunohistochemical analysis of proliferation (PCNA) and DNA damage (γH2AX) in xenografts. The scale bar represents 50 μM. *n* = 5 independent experiments. **H** Body weight changes of xenografts treated with olaparib and cisplatin individually or in combination. Data are shown as the mean ± SD. *P* values are derived from two-way ANOVA.

HRD status provides significant improvement over clinical variables in identifying tumors with an increased likelihood of response to platinum-based neoadjuvant therapy. By comparing the HRD score of OS with pan-cancer data published in TCGA cohort^49^, we found that the HRD score of OS ranked second only following ovarian cancer (Fig. 7A), suggesting PARP targeting therapy may be a promising strategy in OS. Moreover, as described above, nearly 80% of patients in iCluster3 were HRD positive. These patients may be more sensitive to platinum-based neoadjuvant chemotherapy, which could be the main reason for the difference in clinical prognosis between iCluster3 and iCluster4. Genomic CNAs and somatic mutations involved in the HR pathway are labeled (Fig. 7B).

As PARP inhibitors are valuable options for patients with recurrent ovarian cancer in different stages^50–52^, we reasoned that HRD-positive OS may also benefit from PARP inhibitors. Two primary OS cell cultures from PDXs, SA4103 (HRD score: 73, BRCA2: C8318A) and SA4061 (HRD score: 38), were treated with cisplatin or olaparib for 72 h. Cell proliferation was estimated using a CellTiter-Glo luminescent cell viability assay. The results demonstrated that either cisplatin or olaparib induced a more potent anti-proliferative response in the HRD-positive OS cell line SA4103 than in the HRD-negative OS cell line SA4061 or the OS cell line 143B (Fig. 7C). Subsequently, we tested the combinatory effects of cisplatin with olaparib on inhibiting the viability of HRD-positive OS cells and detected a synergistic inhibitory effect (combination index < 1) between cisplatin and olaparib (Fig. 7D).

We confirmed the synthetic lethal effect of cisplatin plus olaparib in vivo using a *BRCA2* mutant OS PDX model, with genetic characteristics of the HRD-positive subtype (SA4078, HRD score: 70, BRCA2: C8318A). Our results revealed that the combination of olaparib and cisplatin led to significantly more shrinkage of flank tumors compared with either single agent (Fig. 7E, F). Furthermore, we measured the induction of cisplatin and olaparib on cell proliferation and the DNA damage pathway in vivo. The results indicated that olaparib alone or in combination with cisplatin significantly inhibited the proliferation of tumor and induced significant DNA damage (Fig. 7G). There was a slight reduction in mouse body weight during treatment, suggesting that the toxicity of the combination regimen was tolerable (Fig. 7H). Collectively, these data demonstrate that PARP inhibition increases lethality induced by cisplatin both in vitro and in vivo.

## Discussion

In recent years, genomic analysis of OS has expanded our knowledge of this genetically complex malignancy^53^. However, no breakthrough has been made in improving the overall survival of OS^54^. One major obstacle is the heterogeneity of OS, meaning that a single therapy could not be successful for all patients. In this study, we characterized an integrated analysis of somatic mutations, copy number alterations, DNA methylation and mRNA expression profiles of 121 OS patients, and this multi-platform dataset provided the most comprehensive atlas of OS genomic, epigenomic and transcriptomic abnormalities to date.

We demonstrated that the mutation spectrum of OS was largely caused by defective DNA repair. Growing evidence indicates that HRD status remains a strong predictor of clinical benefit from PARP inhibitors, which further expands the application prospects of PARPi for OS. Consistent with previous results^10^, we confirmed that CNAs rather than somatic mutations may be the dominant mechanism in OS oncogenesis and development. Of those, *MYC* amplification was the most prominently altered focal CNA. Previous studies demonstrated that the *MYC* oncogene appears to play a role in immunogenicity by creating an environment of immune privilege for the tumors themselves, and *MYC*-targeted therapy induced tumor cell immunogenicity in stimulating host immunity^55^. This may be an important reason for the failure of previous immunotherapy clinical trials for OS. Other significant CNAs involved in several potentially actionable genes, such as *CCNE1, PDGFRA, MDM2, CDK4* and *CDKN2A/B*, need further exploration to determine their therapeutic potential.

Both platform-specific and integrative clustering were carried out to identify the molecular subtypes of OS. We adopted the integration of multi-omics information to achieve more comprehensive subtyping. Surprisingly, we uncovered 4 robust subtypes corresponding to varied clinical prognosis and distinct molecular features. Of note, not only did the genomic features match the clinical prognosis, but there was a strong concordance in subtype assignments between clustering based on individual platforms and iCluster. We also demonstrated the potential therapeutic targets contributing to OS pathogenesis, and offered corresponding therapeutic strategies.

iCluster 1-2 were characterized by elevated immune-related signaling pathways. Notably, the *VEGFA* signaling pathway was activated in these two subgroups. The main challenge in immune therapy was using immune checkpoint inhibitors (ICIs) to noninflamed tumors, and overcoming drug resistance driven by the immune-suppressive microenvironment. *VEGF* results in immune suppression by directly suppressing antigen-presenting cells (APCs) as well as immune effector cells or by augmenting the effects of regulatory T cells (Treg) and tumor-associated macrophages (TAMs). These suppressive immune cells can also drive angiogenesis, creating a vicious cycle of impaired immune activation^56^. The combination of ICIs and anti-*VEGF* therapy has entered the clinic, indicating the potential benefit from this drug combination strategy for iCluster 1–2 patients.

Higher immune infiltration within the tumor is typically associated with a better clinical prognosis^57^, however, we found different clinical prognosis between iCluster1 and iCluster2. Through dissection of intratumoral TCR clones and immune state, iCluster1 (immune-activated) was further distinguished from iCluster2 (immune-suppressed). This indicates that iCluster2 patients may benefit most from ICIs or combination strategies of ICIs and ani-*VEGF* therapy.

Conversely, iCluster 3–4 possessed low immune signatures but higher proliferative activities. Of those, iCluster3 was predominantly composed of HRD-positive patients (84.2%, HRD score > 42), with the highest tumor purity and genomic instability. This suggests that patients in iCluster3 may be preferentially considered for DNA-damaging chemotherapy. In fact, patients with high HRD scores were apt to have a favorable prognosis in platinum-based neoadjuvant chemotherapy^58^, which was verified in our results.

For the most lethal subtype, iCluster4 was characterized by *MYC* amplification and overexpression. *MYC* amplification has been associated with different types of solid tumors, such as neuroblastoma and medulloblastoma^59–61^, while *MYC* overexpression was a defining feature of the most aggressive medulloblastoma subtype^62,63^. Our study highlights the significant roles of *MYC* in iCluster4 during OS progression. Direct or indirect anti-*MYC* agents might be the optimized solution for this subtype, including BET inhibitors, mTOR inhibitors and AURORA kinase inhibitors.

In summary, we provide a genomic, epigenomic and transcriptomic landscape and uncover four distinct molecular subtypes of OS by integrated analytic approaches. Our findings provide important insights into the biology of OS, and can potentially facilitate the development of therapeutic options for this deadly disease.

## Methods

### OS patient sample collection

This study was approved by the Institutional Research Ethics Committee of Shanghai General Hospital, and all participants involved in this study provided written informed consent. Surgical tumor tissues and blood samples from a cohort of 121 OS patients were initially enrolled in the SGH-OS cohort. All patients underwent MAP (methotrexate, doxorubicin, and cisplatin) neoadjuvant chemotherapy and curative resection from February 2011 to August 2019 at Shanghai General Hospital. Tissue samples were collected within 30 min post operation and snap-frozen in liquid nitrogen. Blood samples were collected the day before surgery. Tumors were graded according to the Enneking staging system, and histological diagnoses were established according to the 2020 WHO criteria by two independent pathologists. Overall survival was defined as the time from diagnosis to death. Follow-up of a total of 121 patients was completed on December 31, 2019.

### Generation of the OS PDX model

Fresh tumor specimens were obtained following surgical resection at Shanghai General Hospital. Approximately 100 mg of tissue was placed in a 15 ml polypropylene tube with serum-free DMEM and transferred to the laboratory on wet ice. After washing and cutting, 3–5 mm tumor fragments were implanted into the flanks of NSG mice. Mice were randomized into DMSO or treatment groups when tumors reached approximately 100 mm^3^. The tumor size and body weight of the mice were measured 2 times per week, and the tumor volume was calculated as length × width^2^ × 0.5 mm^3^. All procedures for consideration of animal welfare were reviewed and approved by the ethical committee of the Shanghai General Hospital Animal Care and Use Committee.

### DNA/RNA extraction, quantification and qualification

Genomic DNA from tumor and matched white blood cell samples was isolated using a DNeasy Blood & Tissue Kit (Cat# 69506, QIAGEN, GmBH, Germany) according to the manufacturer’s protocol. DNA was quantified by a Qubit DNA Assay Kit and a Qubit 2.0 Fluorometer (Life Technologies) and the integrity was assessed by 1% agarose gel electrophoresis. A total of 101 tumor samples were used for RNA extraction with TRIzol reagent (Invitrogen). RNA degradation and contamination were monitored on 1% agarose gels; RNA concentration was measured using a Qubit RNA Assay Kit and a Qubit 2.0 Fluorometer (Life Technologies); RNA integrity was assessed using the RNA NanoDrop 8000 Assay Kit and the Agilent 2100 Bioanalyzer system (Agilent Technologies). Samples that passed quality control were sent for genomic characterization.

### DNA/RNA sequencing

#### WES

WES libraries were prepared and captured using the SureSelect Human All Exon V6 kit (Agilent Technologies) following the manufacturer’s instructions. A total of 100 ng of each DNA sample based on Qubit quantification was fragmented into 250~300 bp fragments on a Bioruptor Plus sonication system (Diagenode, Lie’ge, Belgium). Sheared DNA was used to perform end repair, A-tailing and adapter ligation with an Agilent SureSelectXT Library Prep Kit (Agilent Technologies, Santa Clara, CA, USA) according to the manufacturer’s protocol. Then, 750 ng of prepared DNA in a volume of 3.4 ml was captured using Agilent SureSelect Human All Exon V6 (Agilent Technologies) probes, followed by amplification of the captured library with indexing primers. Quality control was performed using an Agilent 2100 Bioanalyzer (Agilent Technologies) with a DNA chip. After quantification with a Qubit 3.0 fluorometer (Invitrogen, Carlsbad, CA, USA), the libraries were sequenced on an Illumina Nova 6000 platform (Illumina Inc., San Diego, CA, USA).

#### RNA sequencing

RNA library preparation was performed as described in the Illumina TruSeq Stranded Total RNA LT sample preparation kit with RiboZero Gold (Illumina Inc., San Diego, CA, USA). Libraries were prepared on an Agilent Bravo Automated Liquid Handling System. Quality control was performed at every step, and the libraries were quantified using a TapeStation system. The mRNA was fragmented to an average insert size of 200–400 bp at 94 °C for 4 min. The cleaved RNA fragments were copied into first-strand cDNA using reverse transcriptase (Invitrogen, Carlsbad, CA, USA) and random primers. The first-strand cDNA was converted into double-stranded DNA in the presence of dUTP. The incorporation of dUTP in second-strand cDNA synthesis quenches the second strand during amplification, thus improving the strand specificity of the library. These cDNA fragments were subjected to the addition of a single ‘A’ base and subsequent ligation of the adapter. The products were purified and enriched via PCR to generate the final library. Quality control was performed at every step, and the libraries were quantified using a TapeStation system. Indexed RNA-seq libraries were then sequenced using the Illumina Nova 6000 platform (Illumina Inc., San Diego, CA, USA).

### Copy number array

Sample DNA was digested by the restriction enzymes NspI, and adapters were ligated to the fragment DNA to perform PCR amplification. Amplified DNA was labeled and further fragmented using an Affymetrix OncoScan array kit and reagent kit bundle, (Cat# 901835, Affymetrix, Santa Clara, CA, US) following the manufacturer’s instructions to obtain biotin-labeled DNA. Hybridization buffers were prepared, and array hybridization was performed at 49 °C in a Hybridization Oven (Cat# 00-0331-220V, Affymetrix, Santa Clara, CA, US). After 16 h of hybridization, arrays were washed in a Fluidics Station (Cat# 00-0079, Affymetrix, Santa Clara, CA, US) according to the manufacturer’s instructions. Arrays were scanned by a GeneChip® Scanner 3000 (Cat# 00-00212, Affymetrix, Santa Clara, CA, US) and Command Console Software 3.1 (Affymetrix, Santa Clara, CA, US) with default settings.

### DNA methylation array

Genomic DNA (gDNA, ≥500 ng) was bisulfite converted using a Zymo EZ DNA Methylation-Gold kit (Zymo Research,Irvine, CA) according to the manufacturer’s instructions. The amount of bisulfite-converted DNA as well as the completeness of bisulfite conversion for each sample were assessed using a panel of MethyLight-based quality control assays. Bisulfite-converted DNAs were whole-genome amplified, enzymatically fragmented, and then hybridized overnight to Infinium EPIC BeadChip arrays (Illumina, San Diego, CA) following the manufacturer’s protocol. The Infinium EPIC array is a genome-wide DNA methylation technique that quantitatively detects over 850,000 (850 K) CpG sites at single nucleotide resolution. Adenine and thymine nucleotides are labeled red, while cytosine nucleotides are labeled green. Arrays were scanned using the Illumina iScan system to produce IDAT files.

### WES data analysis

#### Data processing

The exome sequencing reads after quality control were aligned to the UCSC hg19 reference sequence with Burrows-Wheeler Aligner (bwa mem, v0.7.17). PCR duplicates were removed by Picard (v2.18.11), and the BAM files were then indexed by Samtools (v1.9). Base quality score recalibration was performed by the BaseRecalibrator and ApplyBQSR tools from the Genome Analysis Toolkit (GATK, v4.0.7.0) according to GATK best practices^64^.

#### Mutation calling and filtering

Somatic variants including single nucleotide variants (SNVs) and small indels were detected using Mutect2 in GATK on processed exome data of tumor and matched non-tumor normal samples. Annotation of variants was carried out by Annovar (v2019/04) on the Refseq gene model^65^. Variants in the non-coding regions (upstream, downstream, intergenic, intronic, ncRNA, UTR5, UTR3, etc.) were excluded from the analyses. Germline variants were filtered by using the 1000 Genomes, Exome Aggregation Consortium, NHLBL Exome Sequencing Project (ESP6500) and Genome Aggregation Database (gnomAD). A more stringent downstream filter was applied to obtain high quality somatic variants with the following criteria: a minimum of 8X coverage; Variant Allele Fraction (VAF) ≥ 4% and at least 4 variant supporting reads in the tumor sample, and VAF < 1% in the non-tumor sample; strand bias ≤ 0.95.

Germline variant calling was carried out by using HaplotyperCaller from GATK. Annotation of germline mutations was performed by Annovar (v2019/04) on the Refseq gene model and variants in the non-coding regions were then removed. Pathogenic or likely pathogenic germline variants determined by InterVar^66^ were used for further analysis. KEGG enrichment analysis of germline mutation genes was performed by using the database for annotation, visualization and integrated discovery (DAVID) (https://david.ncifcrf.gov/).

#### Significantly mutated gene identification

The filtered somatic mutations above, including SNVs and indels were further used to identify significantly mutated genes by MutSigCV (v1.4) with default parameters^67^. A false discovery rate (FDR) q value ≤ 0.01 was used as the threshold to determine significantly mutated genes (SMGs).

#### Mutation signature analysis

Somatic mutations are the consequence of multiple mutational processes, including the intrinsic slight infidelity of the DNA replication machinery, exogenous or endogenous mutagen exposures, enzymatic modification of DNA and defective DNA repair. Different mutational processes generate unique combinations of tri-nucleotide mutational contexts, termed “Mutational Signatures”. In this study, the 96 mutational contexts from 107 samples were jointly extracted based on six base substitutions (C > A, C > G, C > T, T > A, T > C, and T > G) within 16 possible combinations of neighboring bases for each substitution using maftools (v2.4.10)^68^. A non-negative matrix factorization (NMF) approach^17^ was implemented to infer the number of mutational signatures given the 96 by 107 mutation matrix. Four signatures were determined based on the cophenetic metric and then compared with the thirty mutational signatures in COSMIC (v3) using cosine similarity to identify the mutation signatures in the SGH-OS cohort.

#### Tumor mutation burden (TMB) analysis

TMB was defined as the number of somatic mutations in the coding region and emerged as a promising biomarker for the prediction of immunotherapy response with checkpoint inhibitors. To reduce sampling bias and reflect the reactive tumor state more immunogenically, synonymous base substitutions and short indels were also counted in the calculation^69^. To calculate the TMB, the total number of mutations counted was divided by the size of the coding sequence region (30 Mb, ~180,000 exons)^70^.

#### Tumor purity, ploidy and HRD score estimation

Paired tumor-normal exome sequencing data were used to estimate tumor purity and ploidy by Sequenza (v3.0.0)^71^. The HRD score was calculated as the sum of the three scores by scarHRD (v0.1.0)^72^ using segments produced by Sequenza: telomeric allelic imbalance (TAI score)^73^, loss of heterozygosity (LOH score)^74^ and large-scale state transition (LST score)^75^.

### RNA-seq data analysis

#### Data processing

RNA-seq clean reads were mapped to the human reference sequence (UCSC hg19 assembly) with Ensembl annotation (GRCh37.75) using STAR (v2.6.1a)^76^ with TranscriptomeSAM mode. The resulting bam file was then subjected to the rsem-calculate-expression program in RSEM (v1.2.28)^77^ for gene expression quantification. The raw count for each gene was calculated by using HTSeq (v0.9.1)^78^.

#### Unsupervised clustering of RNA-seq

Transcripts per million (TPM) quantified by RSEM was used to remove genes whose expression value was quantified as zero in more than 75% of all tumor samples (*n* = 101). The 1820 most variably expressed genes were identified using the cutoff of 0.9 quantile of standard deviation (SD) across all tumor samples. The filtered gene quantifications were then log2 transformed and median centered prior to subsequent analysis. Unsupervised consensus clustering was performed by the ConsensusClusterPlus (v1.46.0)^79^ R package using partitioning around medoids (PAM) with 1-Pearson correlation distance and resampling 80% of the items for 1000 repetitions. Clustering of samples and genes was implemented separately with a maximum of *k* = 10 clusters. The optimal number of clusters (*k* = 4) was determined based on the consensus matrix and the delta area of the relative change in the area under the cumulative distribution function (CDF) curve.

#### Differential gene expression analysis

The DESeq2 package (v1.28.1)^80^ in R was applied to identify genes that were differentially expressed in different clusters. Genes with log2 fold change (FC) ≥ 1 and adjusted p value (P adj) < 0.05 were considered to be statistically significant.

#### Gene set enrichment analysis (GSEA) and single sample GSEA (ssGSEA)

GSEA was carried out by GSEA software (v4.0.0)^81^ and the Molecular Signature Database (MSigDB, v7.1) using the Hallmark^82^, KEGG^83^, and BIOCARTA^84^ gene sets. The pre-ranked tool was used for GSEA with the ranked list in descending order by the statistical significance calculated by DESeq2. Single sample GSEA (ssGSEA) was performed by the GSVA R package (v1.36.2)^85^ using the gene sets curated from the three databases mentioned above.

#### Immune infiltration estimations

The Estimation of STromal and Immune cells in MAligant Tumors using Expression data (ESTIMATE) R package (v1.0.13)^86^ was used to infer the fraction of stromal and immune cells in tumor samples. Cell-type Identification By Estimating Relative Subsets Of RNA Transcripts (CIBERSORT) was employed to estimate the abundance of the 22 immune cell types in each sample^87^. Samples with a deconvolution p value less than 0.05 (96/101) were further used. CDR3 sequences of the tumor-infiltrating TCR were inferred by using TCR repertoire utilities for solid tumors (TRUST, v3.0)^88^. The six immune subtypes (C1: wound healing, C2: *IFN-γ* dominant, C3: inflammatory, C4: lymphocyte depleted, C5: immunologically quite, and C6: *TGF-β* dominant) for each sample were identified by using iAtlas (https://www.cri-iatlas.org/) to investigate the immune microenvironment^48^.

### Copy number analysis

#### Somatic copy number alteration detection

The conumee R package (v1.8.0) was applied to calculate somatic copy number alterations (SCNAs) with default parameters based on Illumina’s 850 K methylation array (*n* = 116). For Affymetrix’s OncoScan array, SCNAs were called by Chromosome Analysis Suite (ChAS, v3.3) software (Affymetrix, Inc.), and these SCNAs were used as the validation dataset (*n* = 50). The segmented copy number profiles were used as an input for Genetic Identification of Significant Targets in Cancer (GISTIC 2.0, v2.0.23)^89^ to identify significantly amplified or deleted regions and obtain gene-level estimates of copy number. GISTIC was run with a 0.99 confidence level and other default parameters. Aberrant regions with FDR Q-values ≤ 0.25 were considered significant.

#### Unsupervised clustering of SCNA

Copy number-based clustering was performed using the SCNA dataset based on Illumina’s 850 K methylation array due to its large sample size (*n* = 116). The “Actual Copy Change Given” for each peak of all tumor samples obtained from GISTIC analysis (all_lesions.conf_99.txt file) was used for clustering. Unsupervised hierarchical clustering was carried out in R based on Euclidean distance using Ward’s method by the ComplexHeatmap (v2.4.3) package^90^. We used hierarchical clustering for copy number as it is more stable for copy number segment data. The cluster assignments were generated by cutting the resulting dendrogram.

#### Effects of copy number alterations (CNAs)

CNAs affecting mRNA in either “cis” (within the same aberrant locus) or “trans” (remote locus) mode were visualized by the multiOmicsViz (v1.10.0) package in R. Spearman correlation coefficients and associated multiple test FDR were calculated for 17,242 mRNA-CNA pairs of 91 samples with both mRNA abundance and CNA data. Genes with significant correlations (FDR < 0.01) were subjected to the enrichGO function in the clusterProfiler (v3.16.1) R package^91^ to identify the enriched biological processes of Gene Ontology^92,93^ altered by CNAs.

### DNA methylation data analysis

#### Data processing

The raw IDAT files (two per sample) generated by Illumina Infinium MethylationEPIC BeadChip Array (850 K) were preprocessed by using the minfi (v1.25.1) R/Bioconductor package^94^. Preprocessing steps included background correction, dye bias normalization, calculation of beta values and corresponding p value detection. Probes with a detection *p* value greater than 0.01 in a given sample were deemed not to be statistically significantly different from background and were thus excluded from the analyses. The following filtering criteria were applied: (1) removal of probes designed for sequences on X and Y chromosomes; (2) probes within promoter regions defined as (−1500, +1500) bp of the transcription start sites (TSSs); and (3) probes located in CpG islands.

#### Unsupervised clustering analysis

To minimize the influence of variable tumor purity levels on a clustering result, we dichotomized the data using a beta-value of ≥ 0.3 as a threshold for positive DNA methylation. The top 8000 CpG sites (by standard deviation) that were methylated with that threshold in more than 5% of the tumors were used for clustering analysis. Unsupervised hierarchical clustering was performed using Ward’s method to cluster the distance matrix computed with the Jaccard index. The clusters were assigned by cutting the resulting dendrogram. The heatmap was generated by ComplexHeatmap (v2.4.3) using the original beta values but ordered according to the above clustering procedures.

#### Identification of epigenetically regulated genes

Probes that were located in a promoter region (upstream and downstream 1500 bp flanking regions of TSSs) and CpG islands defined by the UCSC database were selected for this analysis. For genes with multiple probes, median beta values were considered. mRNA expression data were log2 transformed (log2 (TPM + 1)) and used to assess the gene expression levels associated with DNA methylation changes. Correlations between DNA methylation and mRNA abundance were evaluated by Spearman correlation. Correction for multiple testing was performed using the Benjamini–Hochberg method. Genes were considered to be epigenetically regulated with FDR < 0.01 and correlation coefficient ≥ 0.35.

### Multi-omics data analysis

To study the subtypes formed by multiple molecular platforms of OS, we applied iCluster for integrative clustering. The iCluster algorithm formulates the problem of subgroup discovery as a joint multivariate regression of multiple data types with reference to a set of common latent variables, which represent the underlying tumor subtypes^31,32^.

#### Data processing

Three molecular platforms DNA copy number, DNA methylation and mRNA gene expression of all available samples (91 patients) were used for integrative clustering. Data were pro-processed using the following procedures as input to the iClusterPlus R package (v1.22.0)^32^. The segmented data of SCNA were reduced to a set of 5,226 non-redundant regions. For DNA methylation and mRNA gene expression data, the SD was used to select the most variable 8,000 CpG sites and 1,820 genes, respectively, as described above. The mRNA features were log2 transformed, normalized, and scaled before being used as an input to iCluster.

#### Model selection

We ran tune.iClusterPlus with different numbers of possible clusters (*k* = 1–5). The number of clusters equals *k* + 1. For each *k*, the optimal combination of clusters was determined by minimizing the Bayesian information criterion (BIC). The optimal number of clusters was chosen at which the percentage of explained variation levelled off (*k* = 3, 4 clusters). The plotHeatmap function in the iClusterPlus R package was used to generate a heatmap sorted by integrated cluster assignment.

### Functional experiments

#### Immunohistochemistry validation

Formalin-fixed and paraffin-embedded 4 μm sections were stained for immunohistochemistry according to standard protocols. The tissue sections were deparaffinized with xylene and rehydrated in graded ethanol. Following peroxidase blocking and antigen retrieval, the sections were incubated with primary antibodies against CD4 (Cat #25229, 1:100), CD8 (Cat #85336, 1:200), FOXP3 (Cat #320101, 1:200), IDO (Cat #86630, 1:400), PCNA (Cat #13110, 1:10000), Phospho-Histone H2A.X (Cat #9718, 1:480), C-MYC (Cat #18583, 1:200), Di-Methyl-Histone H3 (Cat #2901, 1:200), NSD1 (Cat #LSC286303, 1:400) and PD-L1 (Cat #13684, 1:200) at 4 °C overnight. After washing 3 times with TBST, the sections were developed using a DAB Kit (BD Bioscience), and then the tissues were counterstained with hematoxylin. For tissue section imaging, blinded evaluation was executed independently by two pathologists (Leica Microsystems), and the proportion of immune positive cells was calculated.

#### PDX-derived tumor cell isolation and purification

Xenograft tissue was minced using sterile scalpels and dissociated for an average of 45 min in HBSS, 1 mg/ml collagenase (Roche), 25% BSA fraction V (GIBCO) and 100 U/mL penicillin and streptomycin. This was followed by further dissociation using trypsin (GIBCO). Red blood cell lysis was performed with ammonium chloride-potassium (ACK) buffer (Invitrogen). Cells were filtered through a 40 μm filter and resuspended in a solution containing phosphate-buffered saline (PBS), pH 7.2, and 0.5% bovine serum albumin (BSA). Cells were then labeled with MicroBeads (Miltenyi Biotec) and incubated at 4 °C for 15 min. After incubation, the cell suspension was separated using a magnetic separator. After FACS qualification, human cells were resuspended in DMEM with 10% fetal bovine serum for other in vitro assays.

#### Cell viability assay

For the cell proliferation assay, primary PDCs (5 × 10^3^ cells in 100 μl/well) were plated into 96-well plates and incubated overnight at 37 °C. Then the cells were treated with DMSO or the indicated concentration of olaparib, cisplatin, or the combination of olaparib and cis-platinum for 3 days at 37 °C. After drug treatment, 50 μl of CellTiter-Glo reagent (Promega, Madison, WI, USA) was added to each well. Luminescence was recorded with an EnVision plate reader (Perkin Elmer, Waltham, MA).

#### In vivo therapeutic testing

Two PDX models (*BRCA2* mutation, HRD score > 42; *BRCA2* WT, HRD score < 42) were adopted for in vivo synthetic lethal evaluation. Short tandem repeat (STR) analysis was also used to confirm genotype matching between PDX passages and the corresponding human tumor. Representative H&E and immunohistochemical staining results demonstrated that PDX tumors closely resemble the human tumors from which they were derived. Mice were randomized into DMSO or treatment groups when tumors reached approximately 100 mm^3^ (five 4 weeks NSG mice per group). In the drug treatment groups, mice were given olaparib, cis-platinum, or a combination of olaparib and cis-platinum at the indicated concentrations by p.o. three times per week. In the DMSO group, mice were administered DMSO diluted in PBS. The maximal tumor volume for mouse experiments was 1500 mm^3^, and no mice exceeded this maximum. Mice were maintained on a 12 h light/dark cycle under a constant temperature of 24 ± 2 °C and a relative humidity of 55 ± 5%. All procedures were approved by the ethics committee of the Shanghai General Hospital Animal Care and Use Committee. The maximal tumor burden was not exceeded than that permitted by the ethics committee.

### Statistics and reproducibility

Quantification and statistical analysis methods for each of the various data platforms and for integrated analyses are mainly described and referenced in the respective method subsections.

Associations between and among clinical and molecular data were assessed according to the nature of the data for each pair. Student’s *t* test, analysis of variance (ANOVA), the Wilcoxon rank sum test and the Kruskal–Wallis test were applied to compare categorical variables versus continuous variables. For categorical variables versus categorical variables, Fisher’s exact test was used; and for continuous versus continuous variables, Spearman correlation was used. To account for multiple testing, the *p* values were adjusted to the false discovery rate (FDR) using Benjamini–Hochberg correction. Kaplan–Meier survival curves and log-rank tests were used to compare the overall survival between different clusters. All clinical and molecular statistics were performed using R packages (v4.0.2).

For the in vitro and in vivo assays, all experiments were repeated three times, and the data are expressed as the mean ± SD. The statistical significance of differences was determined by two-way ANOVA. Statistical analysis was performed using GraphPad Prism (v8.02). All statistical tests were two-sided unless otherwise specified. Asterisks define significance levels (*p < 0.05; **p < 0.01; ***p < 0.001, ****p < 0.0001).

### Reporting summary

Further information on research design is available in the Nature Portfolio Reporting Summary linked to this article.

## Supplementary information

Supplementary Information

Reporting Summary

Description of Additional Supplementary Files

Supplementary Data 1-11

## Data Availability

The WES and mRNA sequencing data generated in this study have been deposited in the Genome Sequence Archive in National Genomics Data Center, China National Center for Bioinformation/Beijing Institute of Genomics, Chinese Academy of Sciences database under accession code (GSA: HRA003260). The DNA methylation data reported in this paper have been deposited in the OMIX, China National Center for Bioinformation/Beijing Institute of Genomics, Chinese Academy of Sciences [https://ngdc.cncb.ac.cn/omix/release/OMIX002042]. The HRD score of pan-cancer data was derived from publicly available TCGA data [https://portal.gdc.cancer.gov/]. Data is available under controlled access due to the conditions stipulated in the patient consent process. WES, RNA-seq and DNA methylation data will be made available for academic use only, and will be made available within 2 weeks. Access can be requested through the GSA access committee, but any queries can be directed to Dr Yingqi Hua (yhua@shsmu.edu.cn). The remaining data are available within the Article, Supplementary Information or Source Data file. Source data are provided with this paper.

## Source data

Source Data

## References

[1] Siegel RL, Miller KD, Jemal A (2020). Cancer statistics, 2020. CA Cancer J. Clin..

[2] Misaghi A, Goldin A, Awad M, Kulidjian AA (2018). Osteosarcoma: a comprehensive review. SICOT J..

[3] Benjamin RS (2020). Adjuvant and neoadjuvant chemotherapy for osteosarcoma: a historical perspective. Adv. Exp. Med. Biol..

[4] George S (2019). Developments in systemic therapy for soft tissue and bone sarcomas. J. Natl Compr. Canc. Netw..

[5] Chen X (2014). Recurrent somatic structural variations contribute to tumorigenesis in pediatric osteosarcoma. Cell Rep..

[6] Bousquet M (2016). Whole-exome sequencing in osteosarcoma reveals important heterogeneity of genetic alterations. Ann. Oncol..

[7] Perry JA (2014). Complementary genomic approaches highlight the PI3K/mTOR pathway as a common vulnerability in osteosarcoma. Proc. Natl Acad. Sci. USA.

[8] Tao J (2014). Notch activation as a driver of osteogenic sarcoma. Cancer Cell.

[9] Mirabello L (2020). Frequency of pathogenic germline variants in cancer-susceptibility genes in patients with osteosarcoma. JAMA Oncol..

[10] Sayles LC (2019). Genome-informed targeted therapy for osteosarcoma. Cancer Discov..

[11] Kovac M (2015). Exome sequencing of osteosarcoma reveals mutation signatures reminiscent of BRCA deficiency. Nat. Commun..

[12] Suehara Y (2019). Clinical genomic sequencing of pediatric and adult osteosarcoma reveals distinct molecular subsets with potentially targetable alterations. Clin. Cancer Res..

[13] Behjati S (2017). Recurrent mutation of IGF signalling genes and distinct patterns of genomic rearrangement in osteosarcoma. Nat. Commun..

[14] Bailey MH (2018). Comprehensive Characterization of Cancer Driver Genes and Mutations. Cell.

[15] Sondka Z (2018). The COSMIC Cancer Gene Census: describing genetic dysfunction across all human cancers. Nat. Rev. Cancer.

[16] Chakravarty, D. et al. OncoKB: a precision oncology knowledge base. *JCO Precis Oncol* **2017**, 10.1200/PO.17.00011 (2017). 10.1200/PO.17.00011 PMC5586540 28890946

[17] Gaujoux R, Seoighe C (2010). A flexible R package for nonnegative matrix factorization. BMC Bioinform..

[18] Alexandrov LB (2015). Clock-like mutational processes in human somatic cells. Nat. Genet.

[19] Loibl S (2018). Survival analysis of carboplatin added to an anthracycline/taxane-based neoadjuvant chemotherapy and HRD score as predictor of response-final results from GeparSixto. Ann. Oncol..

[20] Heeke, A. L. et al. Prevalence of homologous recombination-related gene mutations across multiple cancer types. *JCO Precis. Oncol*. **2018**, 10.1200/PO.17.00286 (2018). 10.1200/PO.17.00286 PMC6139373 30234181

[21] Pilie PG, Gay CM, Byers LA, O’Connor MJ, Yap TA (2019). PARP inhibitors: extending benefit beyond BRCA-mutant cancers. Clin. Cancer Res..

[22] Gong X (2017). Genomic aberrations that activate D-type cyclins are associated with enhanced sensitivity to the CDK4 and CDK6 inhibitor abemaciclib. Cancer Cell.

[23] Cancer Genome Atlas, N. (2015). Comprehensive genomic characterization of head and neck squamous cell carcinomas. Nature.

[24] Weinberg DN (2019). The histone mark H3K36me2 recruits DNMT3A and shapes the intergenic DNA methylation landscape. Nature.

[25] Choufani S (2015). NSD1 mutations generate a genome-wide DNA methylation signature. Nat. Commun..

[26] Papillon-Cavanagh S (2017). Impaired H3K36 methylation defines a subset of head and neck squamous cell carcinomas. Nat. Genet.

[27] Liang L, Fang JY, Xu J (2016). Gastric cancer and gene copy number variation: emerging cancer drivers for targeted therapy. Oncogene.

[28] Su D (2019). Identification of predictors of drug sensitivity using patient-derived models of esophageal squamous cell carcinoma. Nat. Commun..

[29] Rappoport N, Shamir R (2019). Multi-omic and multi-view clustering algorithms: review and cancer benchmark. Nucleic Acids Res.

[30] Rappoport N, Shamir R (2019). NEMO: cancer subtyping by integration of partial multi-omic data. Bioinformatics.

[31] Mo Q (2013). Pattern discovery and cancer gene identification in integrated cancer genomic data. Proc. Natl Acad. Sci. USA.

[32] Shen R, Olshen AB, Ladanyi M (2009). Integrative clustering of multiple genomic data types using a joint latent variable model with application to breast and lung cancer subtype analysis. Bioinformatics.

[33] Georg I, Diaz-Barreiro A, Morell M, Pey AL, Alarcon-Riquelme ME (2020). BANK1 interacts with TRAF6 and MyD88 in innate immune signaling in B cells. Cell Mol. Immunol..

[34] Wu YY, Kumar R, Haque MS, Castillejo-Lopez C, Alarcon-Riquelme ME (2013). BANK1 controls CpG-induced IL-6 secretion via a p38 and MNK1/2/eIF4E translation initiation pathway. J. Immunol..

[35] Yeh ES (2013). Hunk negatively regulates c-myc to promote Akt-mediated cell survival and mammary tumorigenesis induced by loss of Pten. Proc. Natl Acad. Sci. USA.

[36] Wu CC (2020). Immuno-genomic landscape of osteosarcoma. Nat. Commun..

[37] Lettieri CK (2016). Progress and opportunities for immune therapeutics in osteosarcoma. Immunotherapy.

[38] Ratti C (2017). Trabectedin overrides osteosarcoma differentiative block and reprograms the tumor immune environment enabling effective combination with immune checkpoint inhibitors. Clin. Cancer Res..

[39] Wedekind MF, Wagner LM, Cripe TP (2018). Immunotherapy for osteosarcoma: Where do we go from here?. Pediatr. Blood Cancer.

[40] Koirala P (2016). Immune infiltration and PD-L1 expression in the tumor microenvironment are prognostic in osteosarcoma. Sci. Rep..

[41] Gomez-Brouchet A (2017). CD163-positive tumor-associated macrophages and CD8-positive cytotoxic lymphocytes are powerful diagnostic markers for the therapeutic stratification of osteosarcoma patients: An immunohistochemical analysis of the biopsies fromthe French OS2006 phase 3 trial. Oncoimmunology.

[42] Cancer Genome Atlas Research Network. Electronic address, w. b. e.Cancer Genome Atlas Research Network (2017). Comprehensive and integrative genomic characterization of hepatocellular carcinoma. Cell.

[43] Spranger S, Bao R, Gajewski TF (2015). Melanoma-intrinsic beta-catenin signalling prevents anti-tumour immunity. Nature.

[44] Rooney MS, Shukla SA, Wu CJ, Getz G, Hacohen N (2015). Molecular and genetic properties of tumors associated with local immune cytolytic activity. Cell.

[45] Chen B, Khodadoust MS, Liu CL, Newman AM, Alizadeh AA (2018). Profiling tumor infiltrating immune cells with CIBERSORT. Methods Mol. Biol..

[46] Lu J (2019). Molecular constraints on CDR3 for thymic selection of MHC-restricted TCRs from a random pre-selection repertoire. Nat. Commun..

[47] Li B (2016). Landscape of tumor-infiltrating T cell repertoire of human cancers. Nat. Genet..

[48] Thorsson V (2018). The immune landscape of cancer. Immunity.

[49] Knijnenburg TA (2018). Genomic and molecular landscape of DNA damage repair deficiency across the cancer genome atlas. Cell Rep..

[50] Coleman RL (2019). Veliparib with first-line chemotherapy and as maintenance therapy in ovarian cancer. N. Engl. J. Med..

[51] Ray-Coquard I (2019). Olaparib plus Bevacizumab as first-line maintenance in ovarian cancer. N. Engl. J. Med.

[52] Moschetta M, George A, Kaye SB, Banerjee S (2016). BRCA somatic mutations and epigenetic BRCA modifications in serous ovarian cancer. Ann. Oncol..

[53] Lucchesi C (2018). Targetable alterations in adult patients with soft-tissue sarcomas: insights for personalized therapy. JAMA Oncol..

[54] Gianferante DM, Mirabello L, Savage SA (2017). Germline and somatic genetics of osteosarcoma - connecting aetiology, biology and therapy. Nat. Rev. Endocrinol..

[55] Wu, X. et al. MYC oncogene is associated with suppression of tumor immunity and targeting Myc induces tumor cell immunogenicity for therapeutic whole cell vaccination. *J. Immunother. Cancer* **9**, 10.1136/jitc-2020-001388 (2021). 10.1136/jitc-2020-001388 PMC7993333 33757986

[56] Rahma OE, Hodi FS (2019). The intersection between tumor angiogenesis and immune suppression. Clin. Cancer Res..

[57] Robertson AG (2018). Integrative analysis identifies four molecular and clinical subsets in uveal melanoma. Cancer Cell.

[58] Jiang YZ (2019). Genomic and transcriptomic landscape of triple-negative breast cancers: subtypes and treatment strategies. Cancer Cell.

[59] Rickman DS, Schulte JH, Eilers M (2018). The expanding world of N-MYC-driven tumors. Cancer Disco..

[60] Bosse KR (2017). Identification of GPC2 as an oncoprotein and candidate immunotherapeutic target in high-risk neuroblastoma. Cancer Cell.

[61] Northcott PA (2017). The whole-genome landscape of medulloblastoma subtypes. Nature.

[62] Northcott PA (2012). Subgroup-specific structural variation across 1,000 medulloblastoma genomes. Nature.

[63] Northcott PA (2012). Medulloblastomics: the end of the beginning. Nat. Rev. Cancer.

[64] McKenna A (2010). The Genome Analysis Toolkit: a MapReduce framework for analyzing next-generation DNA sequencing data. Genome Res..

[65] Wang K, Li M, Hakonarson H (2010). ANNOVAR: functional annotation of genetic variants from high-throughput sequencing data. Nucleic Acids Res..

[66] Li Q, Wang K (2017). InterVar: clinical interpretation of genetic variants by the 2015 ACMG-AMP guidelines. Am. J. Hum. Genet..

[67] Lawrence MS (2013). Mutational heterogeneity in cancer and the search for new cancer-associated genes. Nature.

[68] Mayakonda A, Lin DC, Assenov Y, Plass C, Koeffler HP (2018). Maftools: efficient and comprehensive analysis of somatic variants in cancer. Genome Res..

[69] Truesdell J, Miller VA, Fabrizio D (2018). Approach to evaluating tumor mutational burden in routine clinical practice. Transl. Lung Cancer Res..

[70] Buttner R (2019). Implementing TMB measurement in clinical practice: considerations on assay requirements. ESMO Open.

[71] Favero F (2015). Sequenza: allele-specific copy number and mutation profiles from tumor sequencing data. Ann. Oncol..

[72] Sztupinszki Z (2018). Migrating the SNP array-based homologous recombination deficiency measures to next generation sequencing data of breast cancer. NPJ Breast Cancer.

[73] Hofer M, Pospisil M, Hoferova Z, Weiterova L, Komurkova D (2012). Stimulatory action of cyclooxygenase inhibitors on hematopoiesis: a review. Molecules.

[74] Abkevich V (2012). Patterns of genomic loss of heterozygosity predict homologous recombination repair defects in epithelial ovarian cancer. Br. J. Cancer.

[75] Popova T (2012). Ploidy and large-scale genomic instability consistently identify basal-like breast carcinomas with BRCA1/2 inactivation. Cancer Res..

[76] Dobin A (2013). STAR: ultrafast universal RNA-seq aligner. Bioinformatics.

[77] Li B, Dewey CN (2011). RSEM: accurate transcript quantification from RNA-Seq data with or without a reference genome. BMC Bioinform..

[78] Anders S, Pyl PT, Huber W (2015). HTSeq–a Python framework to work with high-throughput sequencing data. Bioinformatics.

[79] Wilkerson MD, Hayes DN (2010). ConsensusClusterPlus: a class discovery tool with confidence assessments and item tracking. Bioinformatics.

[80] Love MI, Huber W, Anders S (2014). Moderated estimation of fold change and dispersion for RNA-seq data with DESeq2. Genome Biol..

[81] Subramanian A (2005). Gene set enrichment analysis: a knowledge-based approach for interpreting genome-wide expression profiles. Proc. Natl Acad. Sci. USA.

[82] Liberzon A (2015). The Molecular Signatures Database (MSigDB) hallmark gene set collection. Cell Syst..

[83] Kanehisa M, Goto S (2000). KEGG: kyoto encyclopedia of genes and genomes. Nucleic Acids Res..

[84] Rouillard, A. D. et al. The harmonizome: a collection of processed datasets gathered to serve and mine knowledge about genes and proteins. *Database* **2016**, 10.1093/database/baw100 (2016). 10.1093/database/baw100 PMC4930834 27374120

[85] Hanzelmann S, Castelo R, Guinney J (2013). GSVA: gene set variation analysis for microarray and RNA-seq data. BMC Bioinform..

[86] Yoshihara K (2013). Inferring tumour purity and stromal and immune cell admixture from expression data. Nat. Commun..

[87] Newman AM (2015). Robust enumeration of cell subsets from tissue expression profiles. Nat. Methods.

[88] Li B (2017). Ultrasensitive detection of TCR hypervariable-region sequences in solid-tissue RNA-seq data. Nat. Genet.

[89] Mermel CH (2011). GISTIC2.0 facilitates sensitive and confident localization of the targets of focal somatic copy-number alteration in human cancers. Genome Biol..

[90] Gu Z, Eils R, Schlesner M (2016). Complex heatmaps reveal patterns and correlations in multidimensional genomic data. Bioinformatics.

[91] Yu G, Wang LG, Han Y, He QY (2012). clusterProfiler: an R package for comparing biological themes among gene clusters. OMICS.

[92] Ashburner M (2000). Gene ontology: tool for the unification of biology. Gene Ontol. Consort. Nat. Genet.

[93] The Gene Ontology, C. (2019). The Gene Ontology Resource: 20 years and still GOing strong. Nucleic Acids Res..

[94] Aryee MJ (2014). Minfi: a flexible and comprehensive Bioconductor package for the analysis of Infinium DNA methylation microarrays. Bioinformatics.

