# Supplementary material for: Multi-omics analysis identifies osteosarcoma subtypes with distinct prognosis indicating stratified treatment
Source: Nat Commun. 2022 Nov 23;13:7207. doi: 10.1038/s41467-022-34689-5 (PMC9684515; doi:10.1038/s41467-022-34689-5)
Supplement: Supplementary file 1 — Supplementary Information [file 41467_2022_34689_MOESM1_ESM.pdf]

Multi-omics analysis identifies osteosarcoma subtypes with distinct prognosis indicating stratified treatment

Yafei Jiang et al.

Supplementary Information

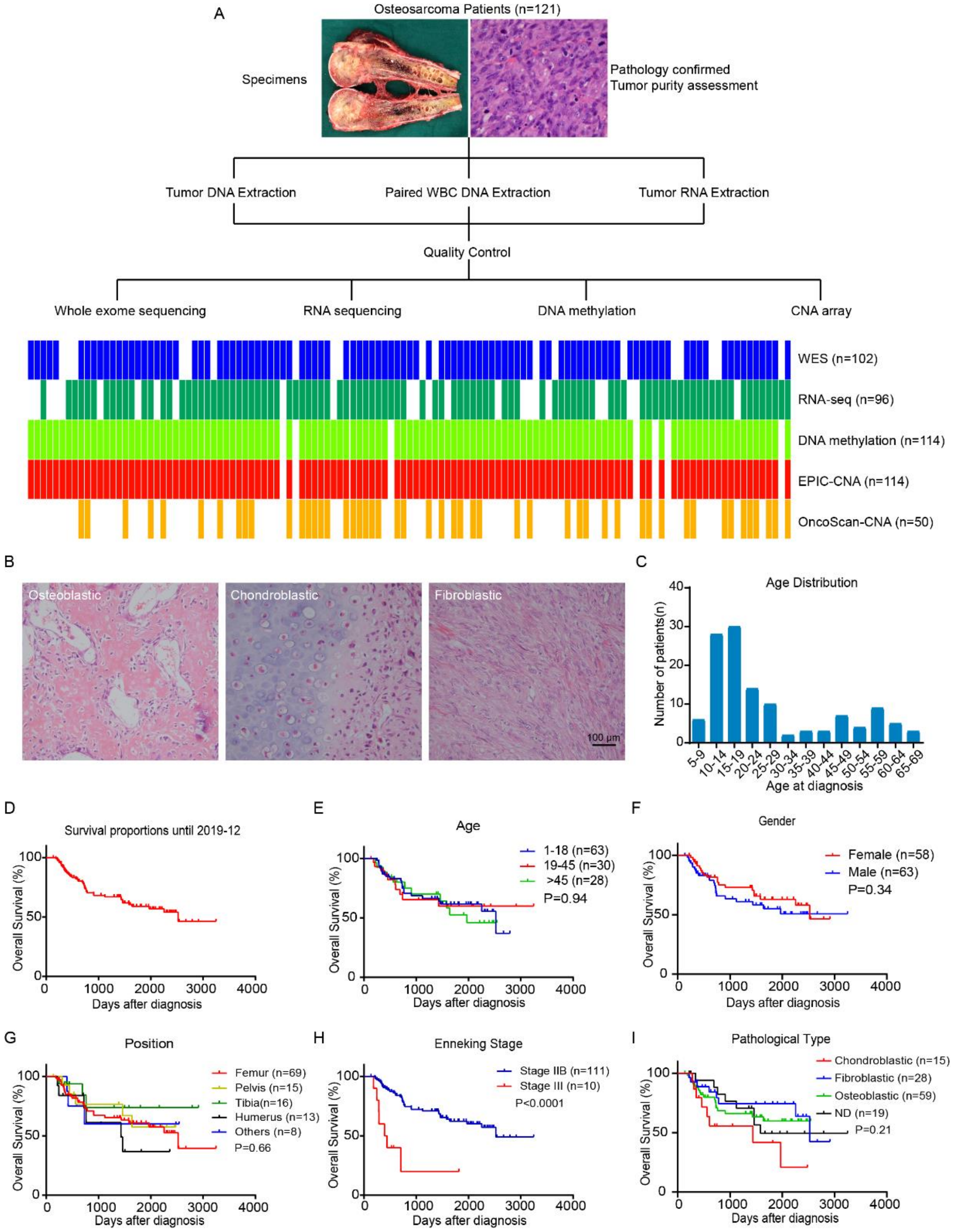

Supplementary Fig. 1. The workflow of the study and the clinical characteristics of the SGH-OS cohort. (A) Frozen tissues from a cohort of 121 patients were obtained for WES (blood also included), RNA-seq, DNA methylation, and DNA copy number array analyses. The tumor purities were assessed by HE staining. Ultimately, 107 WES samples from 102 patients, 101 RNA-seq samples from 96 patients, 116 DNA methylation arrays from 114 patients, and 50 DNA copy number arrays from 50 patients were obtained. (B) Typical pathological subtypes of OS. n = 3 independent experiments. (C) Age distribution of the patients in the SGH-OS cohort. (D) Overall clinical prognosis of the SGH-OS cohort. (E) Overall clinical prognosis of patients among different ages. (F) Overall clinical prognosis of patients of different sexes. (G) Overall clinical prognosis of patients among different tumor positions. (H) Overall clinical prognosis of patients between different Enneking stages. (I) Overall clinical prognosis of patients among different pathological subtypes.

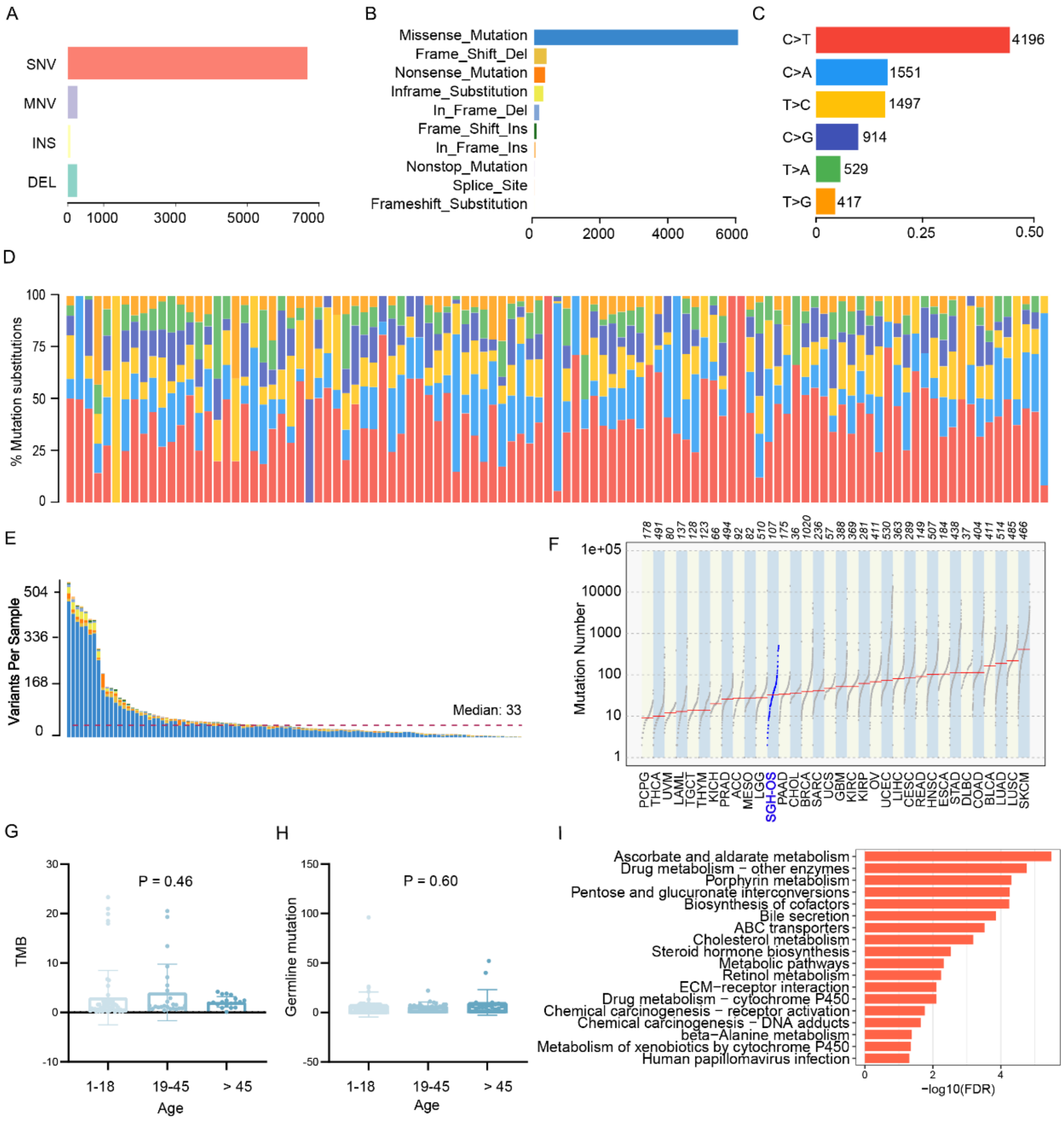

Supplementary Fig. 2. Related to Figure 1. Mutation characteristics of the SGH-OS cohort. (A) Statistics of mutation types. SNV (single nucleotide variants), MNV (multi-nucleotide variants), INS (insertion), DEL (deletion). (B) Statistics of mutation classifications in the SGH-OS cohort. (C) Statistics of mutation substitutions in SGH-OS cohort. (D) Distribution of substitution types in each patient. (E) Distribution of variants per sample. The median number of variants in the SGH-OS cohort was 33. (F) Comparison of the non-synonymous mutation load of the SGH-OS cohort against different tumor types in TCGA, with sample numbers labeled on the top. Tumor types are ordered by their median mutation load. SGH-OS samples are highlighted in blue. (G) Overall TMBs among different age groups. P values are derived from two-way ANOVA. (H) Overall germline mutations

among different age groups. P values are derived from two-way ANOVA. (I) Functional enrichment analysis of germline mutation genes.

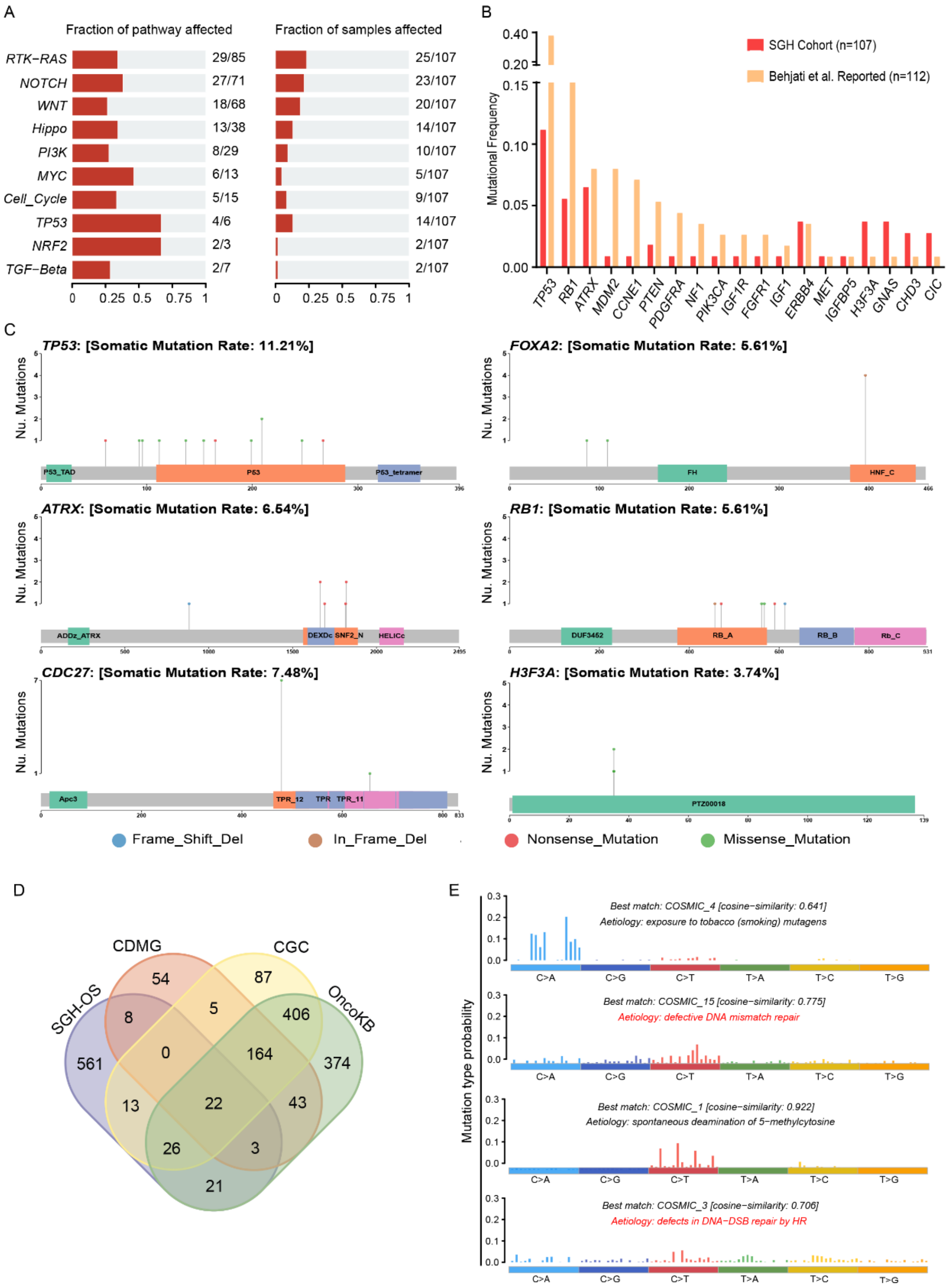

Supplementary Fig. 3. Related to Figure 1. Significantly mutated genes and mutation

signature of the SGH-OS cohort. (A) Oncogenic signaling pathway affected by mutated genes. (B) Comparisons of frequently mutated genes between the SGH-OS cohort and the Behjati cohort. (C) Predicted coding impact by transcript base position and functional domain for selected significantly mutated genes. The height of the lollipop was proportional to the number of mutations at the indicated position. (D) Comparisons of frequently mutated genes (frequency > 3 cases) to cancer genes from CGC, CDMG and OncoKB. (E) Mutation signatures based on 107 samples.

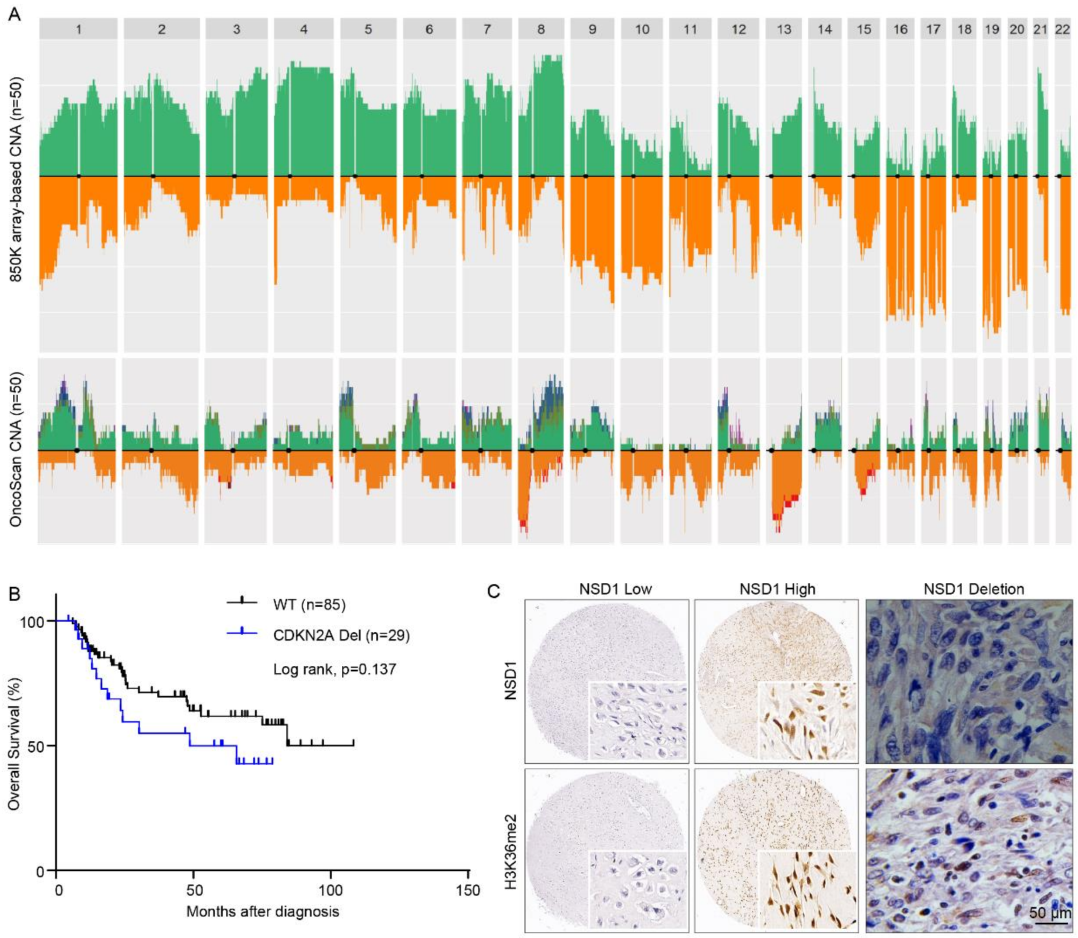

Supplementary Fig. 4. Related to Figure 2. Copy number changes in the SGH-OS cohort. (A) Comparison of copy number changes in 50 cases with a high-resolution OncoScan array and EPIC 850K array. (B) Kaplan-Meier plot of overall survival between CDKN2A deletion patients and WT patients (log-rank test). (C) The expression levels of *NSD1* and H3K36me2 in *NSD1* deletion patients detected by IHC. n = 3 independent experiments.

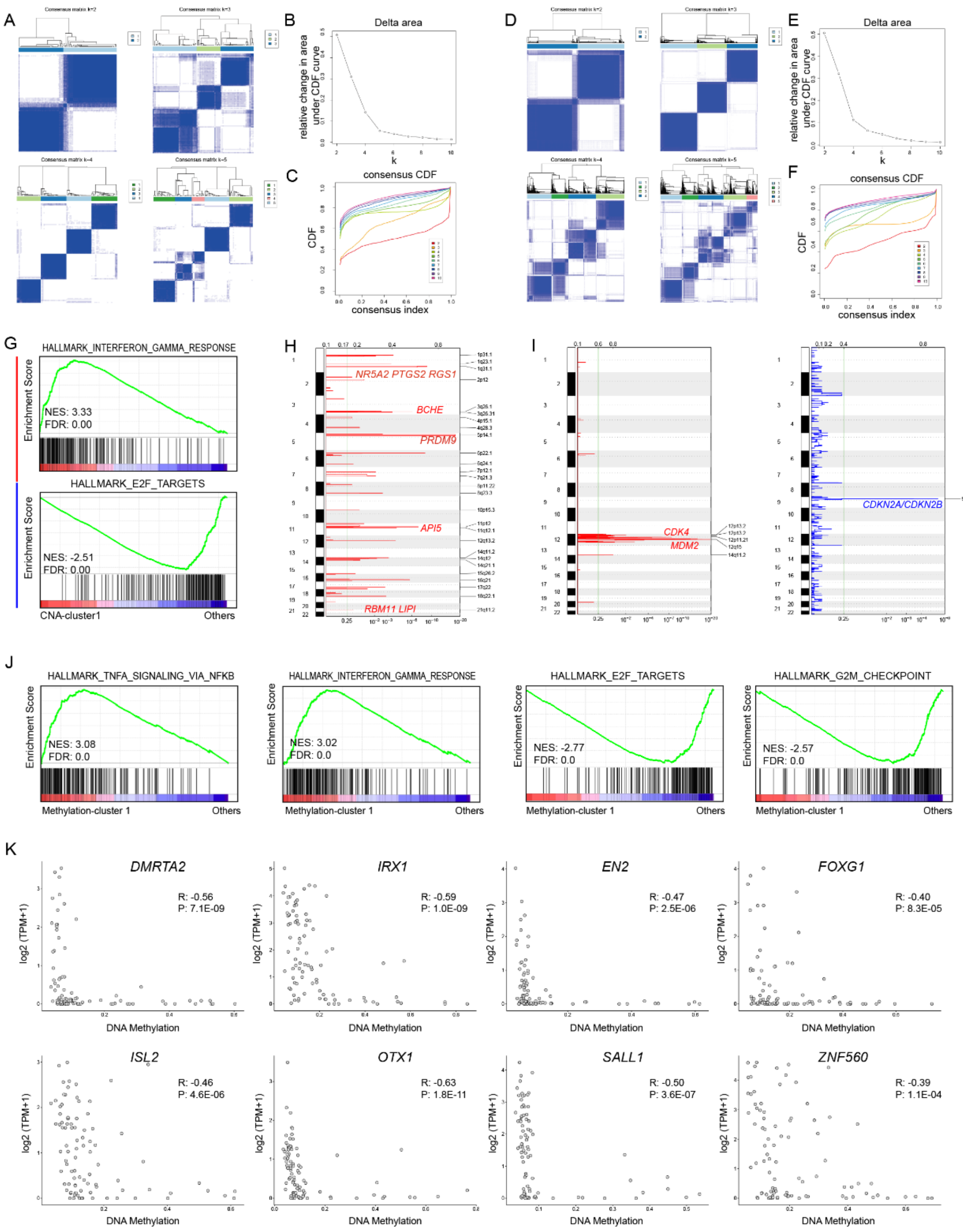

Supplementary Fig. 5. Related to Figure 3. Subgroup characteristics in single platform clustering. (A) Consensus clustering matrices of samples using mRNA data for k = 2 to k = 5. (B) Delta area changes with the number of sample clusters. (C) Consensus empirical cumulative distribution function (CDF) of all given cluster numbers for samples. (D) Consensus clustering matrices of genes using mRNA data for k = 2 to k = 5. (E) Delta area changes with the number of gene clusters. (F) Consensus empirical CDFs of all given cluster numbers for genes. (G) GESA between CNA Cluster1 and others. (H) Regions of focal amplification identified by GISTIC 2.0 in

CNA Clusters 2. (I) Regions of focal amplification and deletion identified by GISTIC 2.0 in CNA Cluster 3. Regions with q values less than 0.25 are considered to be significantly aberrant. (J) GESA between methylation Cluster1 and others. (K) Eight genes for which DNA hypomethylation may contribute to gene abundance.

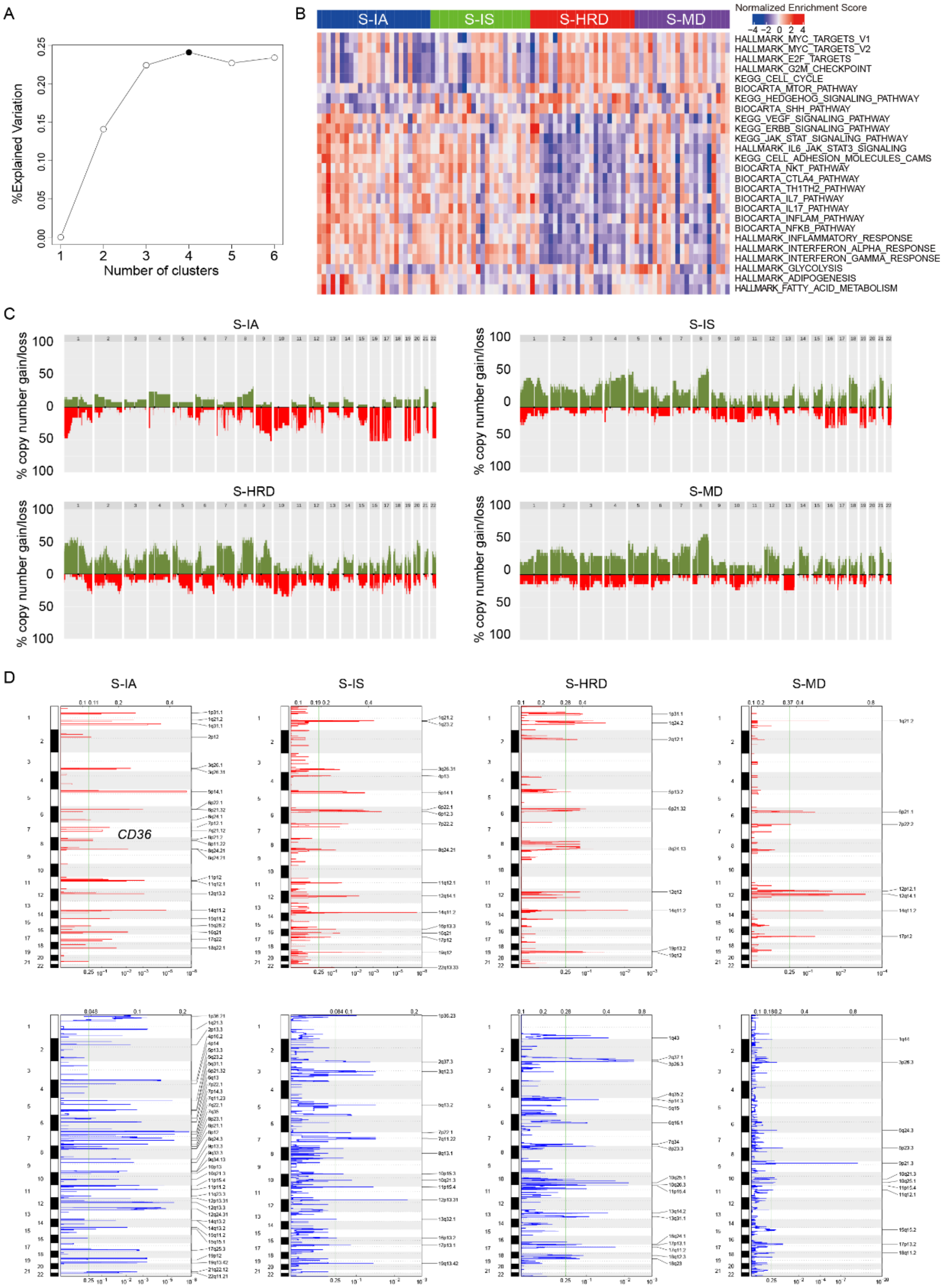

Supplementary Fig. 6. Related to Figure 4. Gene expression and copy-number alterations in each iCluster. (A) Numbers of integrative clusters versus percent of explained variation. (B) Single sample GSEA of differentially activated cancer hallmarks and signaling pathways among the four subtypes. (C) Genome-wide copy-number summary plots for the OS dataset. (D) Recurrent focal amplification (upper panels) and deletion (bottom panels) peaks identified by GISTIC 2.0 according to integrative subgroups.

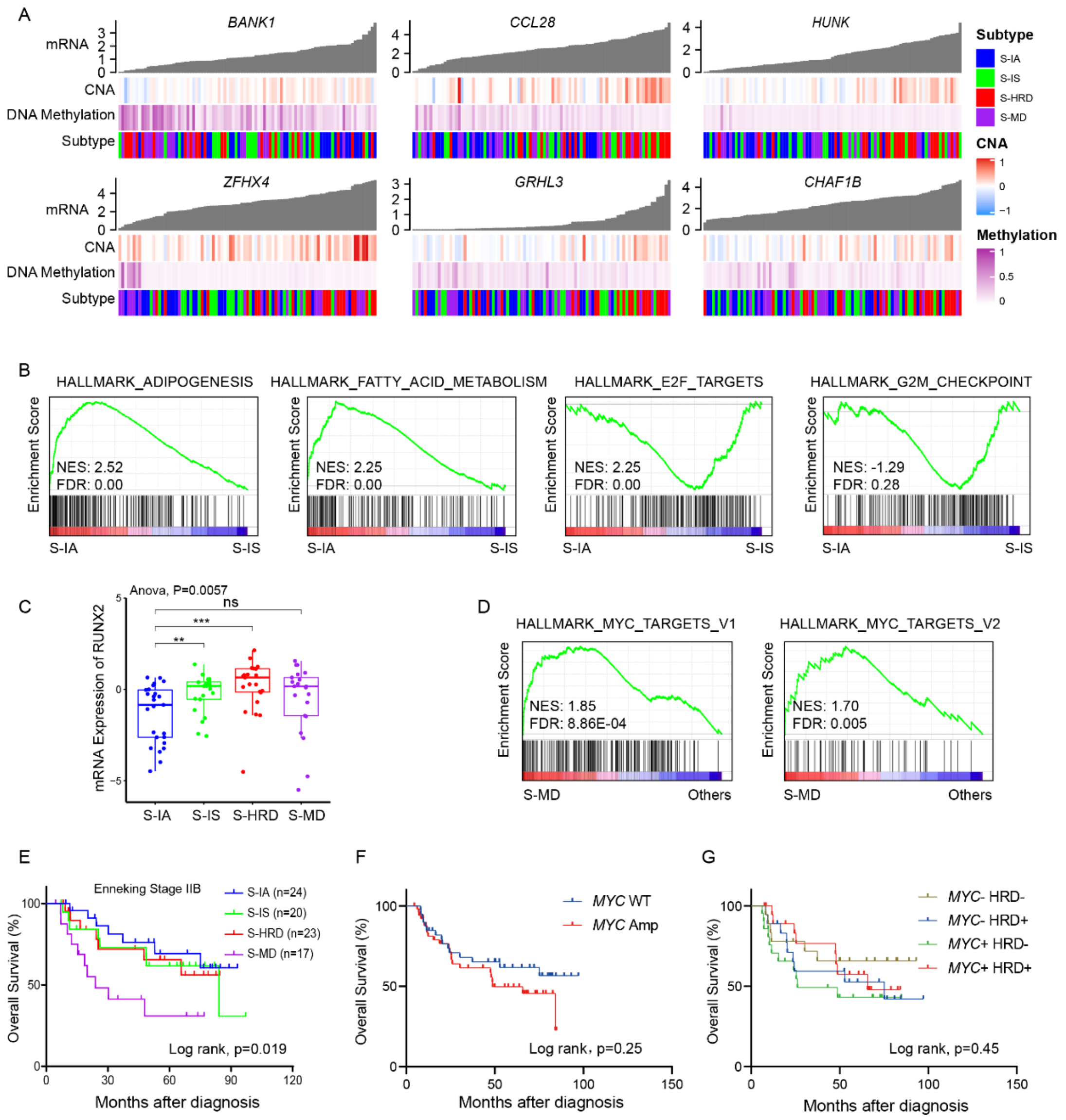

Supplementary Fig. 7. Related to Figure 4. Characteristics of each integrative subtype. (A) Sample-level subtype specific gene expression levels compared to the presence of DNA copy number alterations and DNA methylation. (B) Gene expression level of *RUNX2* in each subtype. The p values were calculated using the two-way ANOVA test. Ns represents non significance. (C

and D) GESA between S-IA and S-IS subtypes. (E) GESA analysis between the S-MD subtype and the others. (F) Kaplan-Meier curves for overall survival of Enneking stage IIB based on integrative subtypes (log-rank test). (G) Kaplan-Meier curves for overall survival based on *MYC* CNAs. (H) Kaplan-Meier curves for overall survival based on combinations of MYC CNAs and HR status.

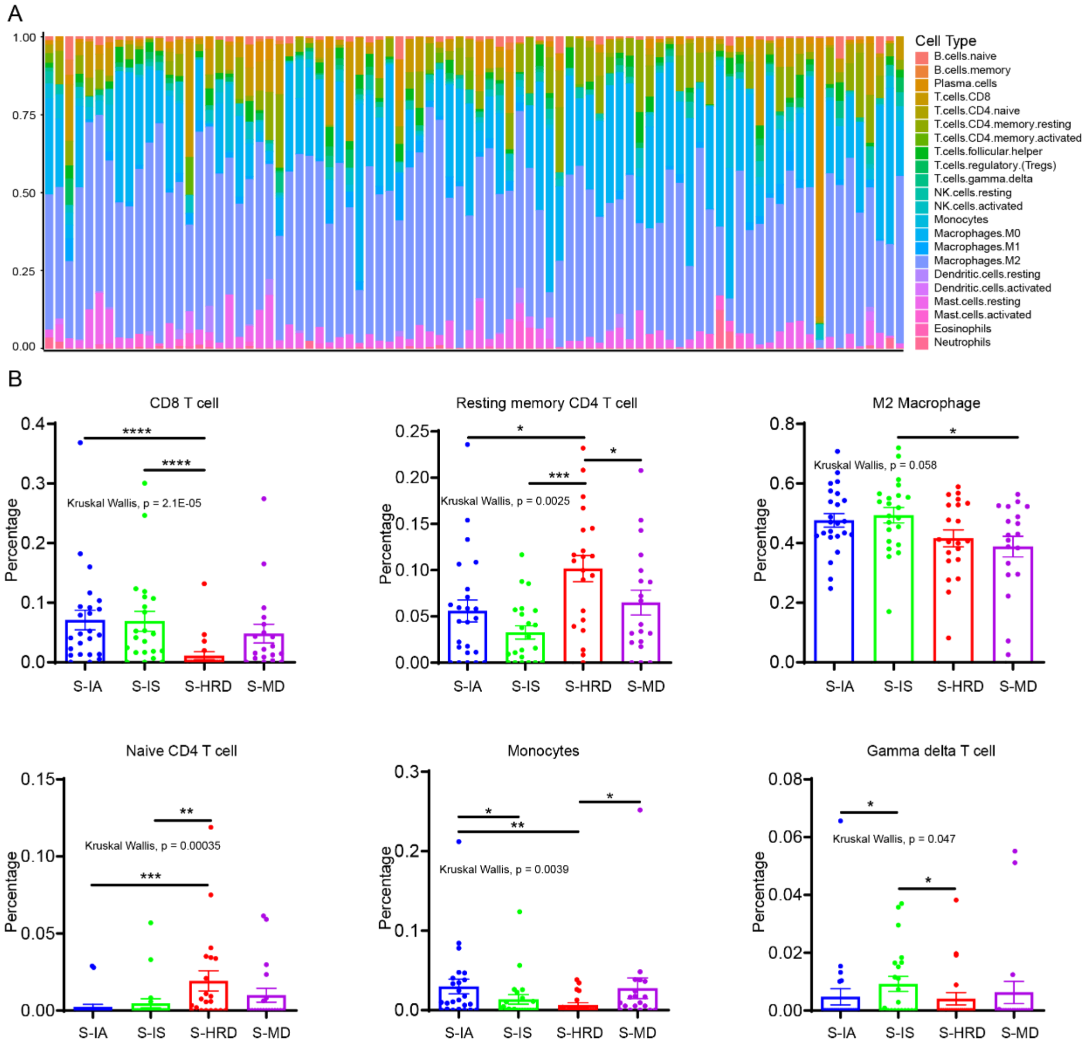

Supplementary Fig. 8. Related to Figure 5. Immune characteristics of the SGH-OS cohort. (A) Relative fractions of different tumor-infiltrating immune cell types for individual patients inferred by the CIBERSORT algorithm. (B) Comparisons of *CD8* T cell, resting memory *CD4* T cell, M2 macrophage, naive *CD4* T cell, monocyte and gamma delta T-cell infiltration levels among different integrative subtypes. The p values were calculated using the Kruskal-Wallis test. Asterisks define significance levels (* $p < 0.05$; ** $p < 0.01$; *** $p < 0.001$, **** $p < 0.0001$).

Supplementary Data 1. Summary clinical parameters for SGH-OS cohort.
Supplementary Data 2 Somatic mutation summary.
Supplementary Data 3. Significant mutation genes.
Supplementary Data 4. Venn diagram of mutation genes.
Supplementary Data 5. Germline mutation genes.
Supplementary Data 6. Copy number alteration "peaks" identified by GISTIC analysis.
Supplementary Data 7. Correlation between CNAs and mRNA expression.
Supplementary Data 8. Correlation between TSS methylation and mRNA expression.
Supplementary Data 9. Subtype specific somatic mutations (Frequency ≥5).
Supplementary Data 10. Gene sets enriched between S-IA and S-IS subtype using GSEA with gene sets in Hallmark.
Supplementary Data 11. CIBERSORT output based on 101 RNA sequencing data.
