# Supplementary material for: Multi-omics analysis identifies osteosarcoma subtypes with distinct prognosis indicating stratified treatment
Source: Nat Commun. 2022 Nov 23;13:7207. doi: 10.1038/s41467-022-34689-5 (PMC9684515; doi:10.1038/s41467-022-34689-5)
Supplement: Supplementary file 2 — Reporting Summary [file 41467_2022_34689_MOESM2_ESM.pdf]

nature portfolio

Corresponding author(s): Yingqi Hua

Last updated by author(s): Aug 31, 2022

Reporting Summary

Nature Portfolio wishes to improve the reproducibility of the work that we publish. This form provides structure for consistency and transparency in reporting. For further information on Nature Portfolio policies, see our Editorial Policies and the Editorial Policy Checklist.

Statistics

For all statistical analyses, confirm that the following items are present in the figure legend, table legend, main text, or Methods section.

n/a	Confirmed	
☐	☒	The exact sample size (n) for each experimental group/condition, given as a discrete number and unit of measurement
☐	☒	A statement on whether measurements were taken from distinct samples or whether the same sample was measured repeatedly
☐	☒	The statistical test(s) used AND whether they are one- or two-sided<br>*Only common tests should be described solely by name; describe more complex techniques in the Methods section.*
☒	☐	A description of all covariates tested
☐	☒	A description of any assumptions or corrections, such as tests of normality and adjustment for multiple comparisons
☐	☒	A full description of the statistical parameters including central tendency (e.g. means) or other basic estimates (e.g. regression coefficient) AND variation (e.g. standard deviation) or associated estimates of uncertainty (e.g. confidence intervals)
☐	☒	For null hypothesis testing, the test statistic (e.g. F, t, r) with confidence intervals, effect sizes, degrees of freedom and P value noted<br>*Give P values as exact values whenever suitable.*
☒	☐	For Bayesian analysis, information on the choice of priors and Markov chain Monte Carlo settings
☐	☒	For hierarchical and complex designs, identification of the appropriate level for tests and full reporting of outcomes
☐	☒	Estimates of effect sizes (e.g. Cohen's d, Pearson's r), indicating how they were calculated

Our web collection on statistics for biologists contains articles on many of the points above.

Software and code

Policy information about availability of computer code

Data collection	Images were captured with an OLYMPUS IX83 microscope
Data analysis	Burrows-Wheeler Aligner (bwa mem, v0.7.17) http://bio-bwa.sourceforge.net<br>Picard (v2.18.11) http://broadinstitute.github.io/picard<br>Samtools (v1.9) http://www.htslib.org/doc/samtools.html<br>GATK4 https://software.broadinstitute.org/gatk/gatk4<br>Annovar (v2019/04) http://www.openbioinformatics.org/annovar/<br>MutsigCV (v1.4) http://software.broadinstitute.org/cancer/<br>maftools (v2.4.10) https://bioconductor.org/packages/release/bioc/html/maftools.html<br>Non-negative matrix factorization (NMF)<br>Sequenza (v3.0.0) http://www.cbs.dtu.dk/biotools/sequenza<br>scarHRD (v0.1.0) https://github.com/sztup/scarHRD<br>STAR (v2.6.1a) https://github.com/alexdobin/STAR<br>HTSeq (v0.9.1) https://htseq.readthedocs.io<br>RSEM (v1.2.28) https://github.com/deweylab/RSEM<br>ConsensusClusterPlus (v1.46.0) https://bioconductor.org/packages/release/bioc/html/ConsensusClusterPlus.html<br>DESeq2 (v1.28.1) https://bioconductor.org/packages/release/bioc/html/DESeq2.html<br>Gene Set Enrichment Analysis<br>(GSEA) v4.0.0 https://www.gsea-msigdb.org/gsea/index.jsp

Single sample GSEA (ssGSEA, v1.36.2) https://www.bioconductor.org/packages/release/bioc/html/GSVA.html
ESTIMATE (v1.0.13) https://bioinformatics.mdanderson.org/estimate/rpackage.html
CIBERSORT https://cibersort.stanford.edu
TRUST (v3.0) https://bitbucket.org/liulab/trust
iAtlas https://www.cri-iatlas.org
TIminer (v1.0.0) https://icbi.i-med.ac.at/software/timiner/timiner.shtml
OptiType (v1.3.2) https://github.com/FRED-2/OptiType
NetMHCpan (v3.0) http://www.cbs.dtu.dk/services/NetMHCpan-3.0
conumee (v1.8.0) http://bioconductor.org/packages/conumee/
Chromosome Analysis Suite (ChAS, v3.3) https://www.thermofisher.com/us/en/home/life-science/microarray-analysis/microarray-analysis-instruments-software-services/microarray-analysis-software/chromosome-analysis-suite.html
GISTIC 2.0 (v2.0.23) https://software.broadinstitute.org/cancer/cga/gistic
ComplexHeatmap (v2.4.3) https://bioconductor.org/packages/release/bioc/html/ComplexHeatmap.html
multiOmicsViz (v1.10.0) https://bioconductor.org/packages/release/bioc/html/multiOmicsViz.html
clusterProfiler (v3.16.1) https://bioconductor.org/packages/release/bioc/html/clusterProfiler.html
minfi (v1.25.1) https://bioconductor.org/packages/release/bioc/html/minfi.html
iClusterPlus (v1.22.0) https://bioconductor.org/packages/release/bioc/html/iClusterPlus.html

For manuscripts utilizing custom algorithms or software that are central to the research but not yet described in published literature, software must be made available to editors and reviewers. We strongly encourage code deposition in a community repository (e.g. GitHub). See the Nature Portfolio guidelines for submitting code & software for further information.

Data

Policy information about availability of data

All manuscripts must include a data availability statement. This statement should provide the following information, where applicable:

- Accession codes, unique identifiers, or web links for publicly available datasets
- A description of any restrictions on data availability
- For clinical datasets or third party data, please ensure that the statement adheres to our policy

The WES, OncoScan CNV array, 850K DNA methylation array and mRNA sequencing data generated in this study have been deposited in the NODE (https://www.biosino.org/node) database under accession code OEP002163 [https://www.biosino.org/node/project/detail/OEP002163.].

Human research participants

Policy information about studies involving human research participants and Sex and Gender in Research.

Reporting on sex and gender	121 OS patients were initially enrolled in our study, of which 58 (47.9%) were female and 63 (52.1%) were male. The effects of age on clinical prognosis was analysis in Supplementary figure 1F with no significant difference. In addition, the age distribution in different subtypes were basically the same.
Population characteristics	The SGH-OS cohort contains the molecular and clinical data of total 121 primary OS patients with mostly Enneking stage IIB and III. All patients underwent MAP (methotrexate, doxorubicin, and cisplatin) neoadjuvant chemotherapy and curative resection from February 2011 to August 2019 at Shanghai General Hospital. Tumors were graded according to the Enneking staging system, and histological diagnoses were established according to the 2020 WHO criteria by two independent pathologists. Follow-up of a total of 121 patients was completed on December 31, 2019. The initial diagnosis age was between 6 to 67 years old, and 63 patients (52.1%) were pediatric or adolescent (under 18 years old, Supplementary Fig. 1C). Of those, 58 (47.9%) were female and 63 (52.1%) were male. The median follow-up was 34.9 months and the overall survival rate was explored. Among all 121 participants, 61 patients (50.4%) had distant metastatic events, 35 patients (28.9%) suffered local recurrence, and 43 patients (35.5%) had died by the last follow-up. The influence of age, sex, tumor position, Enneking stage and pathological type on clinical prognosis was analyzed. Other basic clinical information of the SGH-OS cohort is available in Supplementary Data 1.
Recruitment	Surgical tumor tissues and blood samples from a cohort of 121 OS patients were initially enrolled in the SGH-OS cohort. All donors signed their consent after being fully informed of the goal and characteristics of our study when they were admitted to hospital. However, since the initial diagnosis age was between 6 to 67 years old, pediatric and elderly osteosarcoma may differ in both genomics and clinical features. However, the pathological subtypes were basically the same in different age groups, young and elderly patients was often considered as the same disease, administrated with the similar therapeutic strategies. Taking these patients together will enable unbiased analysis of the heterogeneity of osteosarcoma, and provide different intervention strategies based on the genomic changes. The detail clinical information of these patients could be found in Supplementary Data 1.
Ethics oversight	The experiments performed in this study were approved by the Ethics Committee of Shanghai General Hospital (License No. 2021SQ118). All participants involved in this study provided written informed consent after being fully informed of the goal and characteristics of our study.

Note that full information on the approval of the study protocol must also be provided in the manuscript.

Field-specific reporting

Please select the one below that is the best fit for your research. If you are not sure, read the appropriate sections before making your selection.

☒ Life sciences ☐ Behavioural & social sciences ☐ Ecological, evolutionary & environmental sciences

For a reference copy of the document with all sections, see nature.com/documents/nr-reporting-summary-flat.pdf

Life sciences study design

All studies must disclose on these points even when the disclosure is negative.

Sample size	No statistical method was used to predetermine sample size. 121 osteosarcoma patients were enroled in this study, we identified the subtypes of osteosarcoma by performing unsupervised integrative clustering via iClusterPlus algorithm, which was commonly utilized by The Cancer Genome Atlas (TCGA) consortia. Four molecular subtypes were obtained with similar number of cases, the sample size was sufficient for data source.
Data exclusions	No participants were excluded.
Replication	All experiments were conducted in at least 3 biological replicates and the experimental findings could be confirmed thereby.
Randomization	In in vivo study, Mice were randomized into DMSO or treatment groups when tumors reached approximately 100 mm3. Allocation of other experiments was random.
Blinding	For animal experiments, the investigators were blinded by using lab codes for each sample not providing the genotype of the animal. For bioinformatic analysis, we explored molecular subtyping by performing unsupervised integrative clustering algorithm so the blindness did not affect the results of this study. For IHC analysis, the results were objective indicators, as the results were obtained from multiple random fields.

Reporting for specific materials, systems and methods

We require information from authors about some types of materials, experimental systems and methods used in many studies. Here, indicate whether each material, system or method listed is relevant to your study. If you are not sure if a list item applies to your research, read the appropriate section before selecting a response.

Materials & experimental systems

n/a	Involved in the study	
☐	☒	Antibodies
☐	☒	Eukaryotic cell lines
☒	☐	Palaeontology and archaeology
☐	☒	Animals and other organisms
☒	☐	Clinical data
☒	☐	Dual use research of concern

Methods

n/a	Involved in the study	
☒	☐	ChIP-seq
☒	☐	Flow cytometry
☒	☐	MRI-based neuroimaging

Antibodies

Antibodies used

Rabbit Polyclonal NSD1 Antibody, LSBio, Cat #LSC286303
Rabbit monoclonal PD-L1 (E1L3N®) antibody ,Cell signaling Technology, Cat #13684
Rabbit monoclonal C-MYC (E5Q6W) antibody, Cell signaling Technology, Cat #18583
Rabbit PCNA (D3H8P) antibody, Cell signaling Technology, Cat #13110
Rabbit Phospho-Histone H2A.X (Ser139) antibody, Cell signaling Technology, Cat #9718
Rabbit Di-Methyl-Histone H3 (Lys36) (C75H12) antibody, Cell signaling Technology, Cat #2901
Rabbit monoclonal CD8α (D8A8Y) antibody, Cell signaling Technology, Cat #85336
Rabbit monoclonal CD4 (D7D2Z) antibody, Cell signaling Technology, Cat #25229
Rabbit monoclonal IDO (D5J4) antibody, Cell signaling Technology, Cat #86630
Mouse monoclonal FOXP3 (206D) Antibody, Biolegend, Cat #320101

Validation

Rabbit Polyclonal NSD1 Antibody, LSBio, Cat #LSC286303, validation stated on supplier's website: https://www.lsbio.com/antibodies/nsd1-antibody-aa650-700-ihc-ls-c286303/295843.
Rabbit monoclonal PD-L1 antibody ,Cell signaling Technology, Cat #13684, validation stated on supplier's website: https://www.cellsignal.cn/products/primary-antibodies/pd-l1-e1l3n-xp-rabbit-mab/13684?site-search-type=Products&N=4294956287&Ntt=13684&fromPage=plp&_requestid=2695882.
Rabbit monoclonal C-MYC antibody, Cell signaling Technology, Cat #18583, validation stated on supplier's website: https://

www.cellsignal.cn/products/primary-antibodies/c-myc-e5q6w-rabbit-mab/18583?_=1636799159394&Ntt=18583&tahead=true.
Rabbit PCNA (D3H8P) antibody, Cell signaling Technology, Cat #13110, validation stated on supplier's website: https://www.cellsignal.cn/products/primary-antibodies/pcna-d3h8p-xp-rabbit-mab/13110?_=1636799186218&Ntt=13110&tahead=true.
Rabbit Phospho-Histone H2A.X (Ser139) antibody, Cell signaling Technology, Cat #9718, validation stated on supplier's website: https://www.cellsignal.cn/products/primary-antibodies/phospho-histone-h2a-x-ser139-20e3-rabbit-mab/9718?_=1636799220169&Ntt=9718&tahead=true.
Rabbit Di-Methyl-Histone H3 (Lys36) (C75H12) antibody, Cell signaling Technology, Cat #2901, validation stated on supplier's website: https://www.cellsignal.cn/products/primary-antibodies/di-methyl-histone-h3-lys36-c75h12-rabbit-mab/2901?site-search-type=Products&N=4294956287&Ntt=2901&fromPage=plp&_requestid=2696130.
Rabbit monoclonal CD8α (D8A8Y) antibody, Cell signaling Technology, Cat #85336, validation stated on supplier's website: https://www.cellsignal.cn/products/primary-antibodies/cd8a-d8a8y-rabbit-mab/85336?_=1636799327903&Ntt=85336&tahead=true.
Rabbit monoclonal CD4 (D7D2Z) antibody, Cell signaling Technology, Cat #25229, validation stated on supplier's website: https://www.cellsignal.cn/products/primary-antibodies/cd4-d7d2z-rabbit-mab/25229?site-search-type=Products&N=4294956287&Ntt=25229&fromPage=plp&_requestid=2696224.
Rabbit monoclonal IDO (D5J4) antibody, Cell signaling Technology, Cat #86630, validation stated on supplier's website: https://www.cellsignal.cn/products/primary-antibodies/ido-d5j4e-rabbit-mab/86630?site-search-type=Products&N=4294956287&Ntt=86630&fromPage=plp&_requestid=2696248.
Mouse monoclonal FOXP3 (206D) Antibody, Biolegend, Cat #320101, validation stated on supplier's website: https://www.biolegend.com/en-us/products/purified-anti-human-foxp3-antibody-2897.

Eukaryotic cell lines

Policy information about cell lines and Sex and Gender in Research

Cell line source(s)	Human osteosarcoma cell line 143B were used in this study were purchased from American Type Culture Collection (ATCC, Manassas, VA).
Authentication	The STR analysis were used for cell line authentication.
Mycoplasma contamination	All cell lines tested negative for mycoplasma contamination.
Commonly misidentified lines (See ICLAC register)	This study did not include misidentified cell lines.

Animals and other research organisms

Policy information about studies involving animals; ARRIVE guidelines recommended for reporting animal research, and Sex and Gender in Research

Laboratory animals	Patient tumor fragments were implanted into the flank of the 4 weeks NSG mice for PDX model.
Wild animals	This study did not include wild animals.
Reporting on sex	All animals used in our study were male NSG.
Field-collected samples	This study did not include field-collected samples.
Ethics oversight	All procedures for consideration of participants and animal welfare were reviewed and approved by the ethical committee of the Shanghai General Hospital.

Note that full information on the approval of the study protocol must also be provided in the manuscript.
