# Supplementary material for: Multi-omics analysis identifies osteosarcoma subtypes with distinct prognosis indicating stratified treatment
Source: Nat Commun. 2022 Nov 23;13:7207. doi: 10.1038/s41467-022-34689-5 (PMC9684515; doi:10.1038/s41467-022-34689-5)
Supplement: Supplementary file 3 — Description of Additional Supplementary Files [file 41467_2022_34689_MOESM3_ESM.pdf]

Description of Additional Supplementary Files

Supplementary Data 1. Summary clinical parameters for SGH-OS cohort.

Supplementary Data 2. Somatic mutation summary.

Supplementary Data 3. Significant mutation genes.

Supplementary Data 4. Venn diagram of mutation genes.

Supplementary Data 5. Germline mutation genes.

Supplementary Data 6. Copy number alteration "peaks" identified by GISTIC analysis.

Supplementary Data 7. Correlation between CNAs and mRNA expression.

Supplementary Data 8. Correlation between TSS methylation and mRNA expression.

Supplementary Data 9. Subtype specific somatic mutations (Frequency ≥5).

Supplementary Data 10. Gene sets enriched between S-IA and S-IS subtype using GSEA with gene sets in Hallmark.

Supplementary Data 11. CIBERSORT output based on 101 RNA sequencing data.
